# Synergistic antitumor effects of *astragalus* polysaccharide: a preclinical systematic review and meta-analysis

**DOI:** 10.3389/fphar.2025.1672450

**Published:** 2025-12-18

**Authors:** Ruo Zhang, Qian Yang, Zhi Chen, Jianming Huang, Guonan Zhang

**Affiliations:** 1 College of Clinical Medicine, Chengdu University of Traditional Chinese Medicine, Chengdu, China; 2 Department of Biochemistry and Molecular Biology, Sichuan Cancer Hospital and Institute, Chengdu, China; 3 Department of Gynecologic Oncology, Sichuan Clinical Research Center for Cancer, Sichuan Cancer Hospital and Institute, Sichuan Cancer Center, University of Electronic Science and Technology of China, Chengdu, China

**Keywords:** astragalus polysaccharide, preclinical studies, antitumor, meta-analysis, traditional Chinese medicine

## Abstract

**Background:**

Accumulating evidence suggests that astragalus polysaccharide (APS) may enhance the efficacy of conventional cancer therapies through multiple mechanisms. However, the synergistic effects of APS have not been systematically quantified. This meta-analysis was therefore conducted to quantify these potential synergistic antitumor effects and provide preclinical evidence to inform future clinical trials.

**Methods:**

Following PRISMA 2020 guidelines, we systematically searched ten databases (including PubMed and Web of Science) for preclinical studies from inception to May 2025 using predefined inclusion criteria. Risk of bias was assessed using SYRCLE’s RoB tool. Meta-analyses and subgroup analyses were performed using RevMan 5.4.1, while publication bias was assessed via funnel plots and Egger’s test (Stata 17.0). This systematic review was prospectively registered in PROSPERO (registration number: CRD420251047751).

**Results:**

Forty-one publications (44 independent studies) involving 748 animals were included. APS combination therapy was associated with significant improvements in tumor-related outcomes, including reduced tumor weight and volume, suppressed metastasis, and prolonged survival. Mechanistically, APS co-administration enhanced CD8^+^ T-cell infiltration, increased splenic and thymic indices, modulated cytokine profiles (TNF-α, IL-2, IFN-γ, IL-12, IL-6, IL-10), and reduced PD-1/PD-L1 expression in tumor tissue. Additionally, APS appeared to alleviate chemotherapy-induced nephrotoxicity, as evidenced by lower serum creatinine levels. Subgroup analyses indicated that heterogeneity was partially explained by model type, APS dosing regimen, and combination therapy modality. The certainty of evidence for primary outcomes was rated as low or very low according to the GRADE assessment.

**Conclusion:**

This meta-analysis provides preclinical evidence that APS may serve as an adjunctive agent to enhance the efficacy of conventional cancer therapies. However, given the low certainty of current evidence, further mechanistic studies and well-designed clinical trials are urgently warranted to establish its efficacy and therapeutic role in oncology.

**Systematic Review Registration:**

PROSPERO 2025 CRD420251047751 https://www.crd.york.ac.uk/PROSPERO/view/CRD420251047751

## 1 Introduction

The clinical management of solid tumors increasingly relies on multimodal, precision-medicine-based strategies that integrate surgery, chemotherapy, radiotherapy, targeted therapy, and immunotherapy (National Cancer Institute, 2017; Wagle et al., 2025). Despite significant therapeutic advances, treatment outcomes remain limited by several persistent challenges, including an immunosuppressive tumor microenvironment (TME), intrinsic or acquired drug resistance, and dose-limiting toxicities that compromise treatment continuity and durable responses (Jairam et al., 2019; Liu B. et al., 2024). These limitations have driven increasing interest in adjunctive approaches capable of enhancing antitumor efficacy while mitigating systemic toxicity. Traditional Chinese Medicine (TCM) has attracted growing attention in this context due to its broad immunomodulatory activity and generally favorable safety profile. TCM applies the *Fuzheng Quxie* (扶正祛邪) principle to cancer therapeutics — a paradigm aimed at reinforcing host defense mechanisms *(Zhengqi*, 正气) while eliminating pathogenic factors (*Xieqi*, 邪气). This paradigm posits that effective tumor control requires concurrently enhancing physiological resilience to potentiate the effects of conventional anticancer therapies.

*Astragalus mongholicus* Bunge (AM; Fabaceae), known medicinally as *Astragali Radix* (*Huangqi*), is a foundational herb in TCM with documented use tracing back to the Han dynasty in the *Shennong Bencao Jing* (ca. 200 CE). For millennia, it has been employed to strengthen host defenses by tonifying qi and nourishing blood. Phytochemical studies have identified numerous bioactive constituents in *Astragali Radix*, primarily including polysaccharides, triterpene saponins (astragalosides), flavonoids, and trace alkaloids (Durazzo et al., 2021; Liu Y. X. et al., 2024). Among these, astragalus polysaccharide (APS) is regarded as the principal immunologically active component linking traditional tonifying functions with modern immune regulation research.

Since the 1970s, APS has been shown to modulate humoral and cellular immunity and promote hematopoietic recovery. In 2001, an injectable APS formulation (China NMPA approval Z20040086) was approved for managing chemotherapy-induced leukopenia and immunodeficiency.

Recent mechanistic studies further indicate that APS enhances antitumor immunity by stimulating immune activity in central and peripheral lymphoid organs; activating macrophages, natural killer cells, dendritic cells, and T lymphocytes; and inducing the secretion of antitumor cytokines (Li et al., 2022; An et al., 2022; Zhou L. et al., 2017). APS can also partially reprogram the immunosuppressive tumor microenvironment through multiple pathways, thereby restoring CD8^+^ T-cell effector function and strengthening immune surveillance (Li et al., 2020; Du et al., 2022). These immunomodulatory actions appear to directly target core pathophysiological barriers that limit the efficacy of cancer treatment. Existing clinical studies on APS injection have primarily focused on improvements in cancer-related fatigue (Chen et al., 2012; Wang et al., 2019; Shen et al., 2024; Chang et al., 2025), quality of life (Guo et al., 2012), and select immune parameters (Tsao et al., 2021), whereas few have examined its potential synergistic antitumor effects. Consequently, despite its biological plausibility and early clinical signals, APS has not been broadly integrated into routine oncology practice. This translational gap likely arises from fragmented efficacy data, inadequate cross-model validation, and insufficient mechanistic exploration of its synergistic potential with conventional therapies.

To help bridge this gap, we performed a systematic review and meta-analysis of preclinical studies to: 1) quantify the potential synergistic antitumor effects of APS when combined with conventional therapies; 2) delineate underlying molecular mechanisms through biomarker correlation analysis; and 3) evaluate methodological rigor using SYRCLE’s risk-of-bias tool to inform future preclinical and translational research.

## 2 Methods

This systematic review and meta-analysis was conducted in accordance with the PRISMA 2020 guidelines (Page et al., 2021) and was prospectively registered in PROSPERO (CRD420251047751).

### 2.1 Search strategy

A systematic literature search was conducted across ten electronic databases, including PubMed, Web of Science, Embase, Scopus, ProQuest, Ovid, the China National Knowledge Infrastructure (CNKI), Wanfang Data, VIP, and the China Biology Medicine Disc (CBM). The search timeframe spanned from database inception to May 2025, and the search was restricted to publications in Chinese or English. Medical Subject Headings (MeSH) combined with free-text keywords were used in the English-language databases. The search strategy incorporated the following key terms: 1) Problem: Neoplasms [MeSH], tumor, cancer, carcinoma, malignan*; 2) Population: animal model, animal experiment, mice, mouse, xenograft, tumor-bearing, transplantable tumor; and 3) Intervention: Astragalus polysaccharide, Astragalus polysacharin, APS. In addition, the Chinese databases were searched using the corresponding Chinese terms to ensure comprehensive retrieval of relevant studies.

### 2.2 Inclusion/exclusion criteria

#### 2.2.1 Inclusion criteria

1. Population: Animal models bearing malignant neoplasms.
2. Intervention: Experimental groups administered APS in combination with conventional antitumor therapies.
3. Comparison: Control groups receiving conventional antitumor therapy alone.
4. Outcomes: Studies reporting at least one quantitative outcome related to antitumor efficacy (e.g., tumor weight, tumor volume, survival time, or mechanism-related biomarkers).
5. Study design: Randomized controlled animal studies.
6. Data availability: Sufficient quantitative data to calculate effect sizes (e.g., mean ± SD, sample size, *p*-values).
7. Language: Studies published in Chinese or English.

#### 2.2.2 Exclusion criteria

1. Population: Clinical studies, *in vitro* experiments, or animal models of non-cancer diseases.
2. Intervention: APS monotherapy; crude Astragalus extracts lacking polysaccharide isolation; or multi-herbal formulations containing APS.
3. Control: Studies using no-treatment controls or non-standard antitumor therapies.
4. Outcomes: Studies without quantifiable antitumor outcomes or those reporting only qualitative findings.
5. Study design: Non-original research (e.g., abstracts, reviews, case reports); studies lacking an independent control group; or cross-over designs.
6. Data limitations: Insufficient quantitative data for meta-analysis (e.g., missing means, SDs, or sample sizes).
7. Language: Studies published in languages other than Chinese or English without accessible translations.
8. Other: Duplicate publications or studies with overlapping datasets.

### 2.3 Study selection and data extraction

The study selection process was conducted in two consecutive stages. Initially, two independent reviewers screened the titles and abstracts of all retrieved records for potential eligibility according to predefined inclusion criteria. In the second stage, the same reviewers independently evaluated the full texts of studies that passed the initial screening to determine final eligibility. Any disagreements were resolved through discussion with a third, senior reviewer. A standardized data-extraction form (Excel) was used to record key study characteristics, including intervention details, experimental design, and outcome measures. To ensure objectivity, a predefined decision rule was applied: when multiple measurements were available, the final time point and the highest APS dose were extracted. This *a priori* strategy was used to assess the maximum potential intervention effect, maintain consistency, and minimize *post hoc* selection bias. Numerical data were extracted directly from tables or text; when outcomes were presented only in graphical form, data were digitized using GetData Graph Digitizer software.

### 2.4 Quality assessment

The quality of the included animal studies was assessed using the SYRCLE Risk of Bias (RoB) tool. This tool evaluates ten methodological domains covering six types of bias (e.g., sequence generation, baseline characteristics, blinding). Each domain was rated as “L” (low risk), “H” (high risk), or “U” (unclear risk due to insufficient methodological reporting). Two reviewers independently conducted the assessment, and any disagreements were resolved by consensus with a third reviewer.

### 2.5 Statistical analysis

Meta-analyses were conducted using Review Manager (RevMan) 5.4.1. All analyzed outcomes were treated as continuous variables. Accordingly, we calculated mean differences (MDs) or standardized mean differences (SMDs) with 95% confidence intervals (CIs), selecting the effect size metric based on measurement scale consistency across studies. Heterogeneity was assessed using the I^2^ statistic and Cochran’s Q test, with I^2^ > 50% or Q-test *p* < 0.10 indicating substantial heterogeneity. When heterogeneity was not statistically significant (I^2^ ≤ 50% and *p* ≥ 0.10), a fixed-effects model was applied; otherwise, a random-effects model was used. Subgroup analyses and meta-regression were performed to explore potential sources of heterogeneity. Publication bias was assessed using funnel plot symmetry and Egger’s regression test (*p* < 0.05 considered significant) in Stata 17.0.

### 2.6 Sensitivity analysis

Sensitivity analyses were conducted using the leave-one-out approach, which involved sequentially omitting individual studies to assess the robustness of the pooled estimates. This approach quantified the influence of each study on the overall effect size.

### 2.7 Assessment of publication bias and evidence quality

Publication bias was assessed using funnel plot symmetry and Egger’s regression test in Stata 17.0, and only when ten or more studies were available for a given outcome. When publication bias was indicated (Egger’s test *p* < 0.05), the trim-and-fill method was applied to adjust for potentially missing studies. The difference between the original and trim-and-fill–adjusted effect sizes was calculated to quantify its impact on result robustness. The overall evidence quality was evaluated using the GRADE framework adapted for preclinical studies, considering risk of bias, inconsistency, indirectness, imprecision, and publication bias. Final certainty ratings were categorized as “High,” “Moderate,” “Low,” or “Very Low.”

## 3 Results

### 3.1 Study inclusion

**FIGURE 1:**
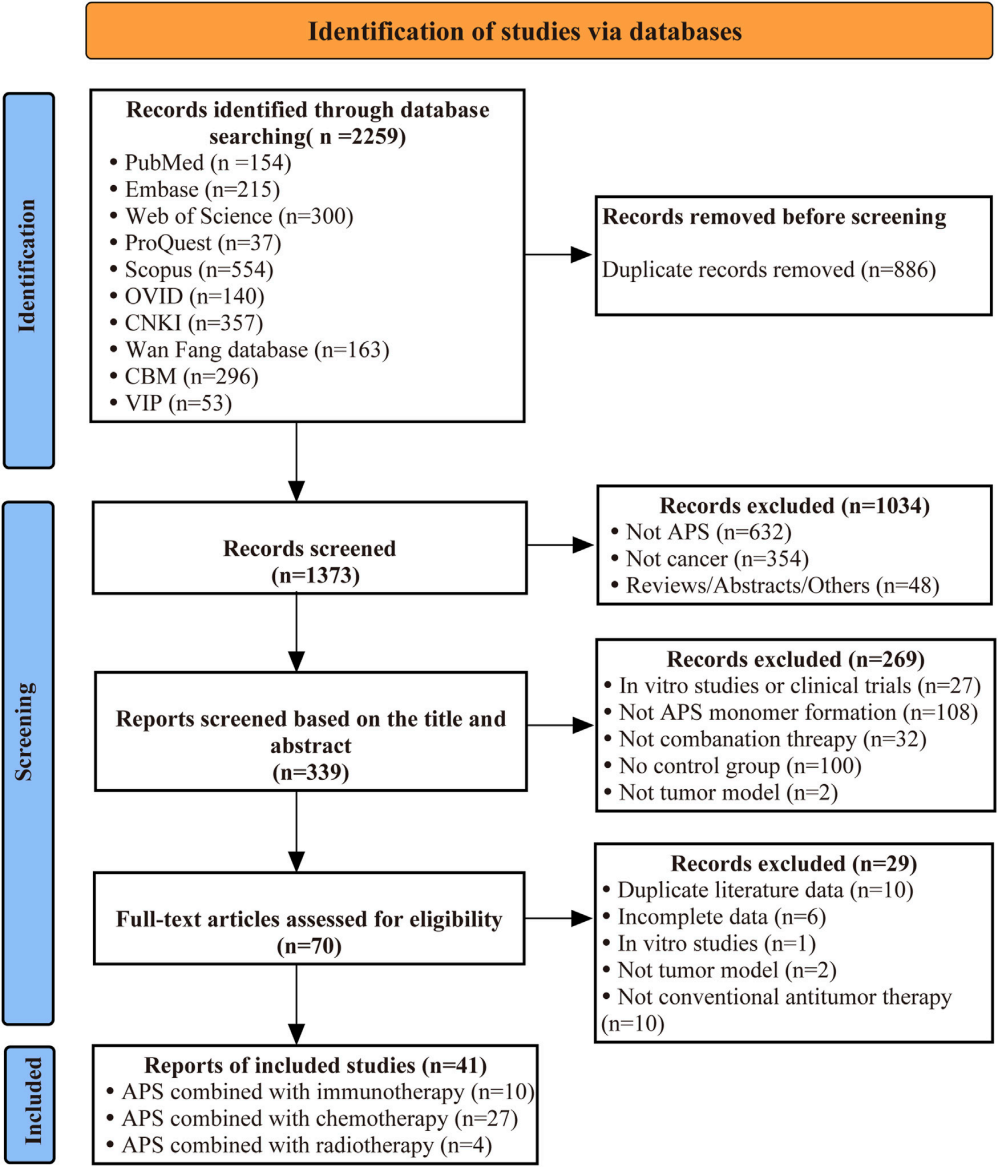
PRISMA flow diagram of study selection.

A total of 2,259 records were identified through database searches. After systematic screening, 41 articles (reporting 44 independent experiments) met the predefined eligibility criteria and were included in the meta-analysis. Figure 1 presents the PRISMA flow diagram illustrating the study selection process.

### 3.2 Characteristics of the included preclinical studies

As detailed in Table 1, the 44 included preclinical studies investigated 11 distinct cancer typesLung cancer (n = 15, 34.1%) and liver cancer (n = 9, 20.5%) were predominant, followed by melanoma (n = 5, 11.4%), breast cancer (n = 4, 9.1%), cervical cancer (n = 3, 6.8%), and colorectal cancer (n = 3, 6.8%). Five additional types—bladder cancer, nasopharyngeal carcinoma, glioma, sarcoma, and pancreatic cancer—were each represented by single studies (n = 1 per type; collectively 11.4%).

**TABLE 1:** Characteristics of studies included in the meta-analysis.

Study	Cancer models	Cell strain and count	Animal species (sex/age or weight)	Sample size	Combination group	APS dosage/administration/frequency	Monotherapy group (dosage/administration/frequency)	Treatment initiation	Followup (days)	Outcome indicator
Huang et al. (2002)	Melanoma	B16, 5 × 10^6^	C57BL/6,F/M, 6–8 w,18–23g	10,10	APS + CD3AK	50 mg/kg, i.p., qd ×3	5 × 10^6^CD3AK cells/mouse, i.p., qd ×3	24 h post-modeling	20	③⑥⑤
Huo (2016)	Cervical	Hela, NR	BALB/c, F, 6–8 w,16–20g	10,10	APS + CIK	200 mg/kg, v., biw ×6	5 × 10^7^ CIK cells/mouse, i.v., biw ×6	6 days post-modeling	21	①
Zhang et al. (2024)	Liver	Huh7, 5 × 10^6^	NOD/SCID, F/M, 6–8 w	5,5	APS + CAR-T	50 mg/kg, p.o., qd ×21	5 × 10^6^ CAR-T cells/mouse, i.v., qd ×1	5 days post-modeling	28	①②⑤⑥
	Liver	HepG2, 2 × 10^6^	NOD/SCID, F/M, 6–8 w	5,5	APS + CAR-T	50 mg/kg, p.o., qd ×21	5 × 10^6^ CAR-T cells/mouse, i.v., qd ×1	5 days post-modeling	28	①②⑥
Sha et al. (2022)	Melanoma	B16, 6 × 10^5^	C57BL/6, F, 6 w	6,6	APS + anti-PD-1	100 mg/kg, p.o., qd ×7	20 mg/kg, i.p., qod ×4	When tumor volume reached 100 mm^3^	7	①②⑥
Lin et al. (2024)	Liver	H22, 3 × 10^6^	BALB/c, M, 6–8 w,18–22g	6,6	APS + anti-PD-1	8 mg/mouse, p.o., qd ×14	200 ug/mouse, i.p., qd ×14	When tumor volume reached 50–100 mm^3^	14	①②⑤⑥⑬
Wang (2024)	Lung	LLC, 2 × 10^6^	C57BL/6, M, 6–8 w,18–22g	5,5	APS + anti-TIGIT	100 mg/kg, i.p., qd ×14	5 mg/kg, i.p., qod ×7	7 days post-modeling	14	①②⑥⑬
Liu et al. (2024b)	Lung	LLC, 2 × 10^6^	C57BL/6, M, 6–8 w	10,10	APS + anti-PD-L1	80 mg/kg, i.p., qd ×14	200 ug/mouse, i.p., q4d ×4	2 days post-modeling	14	①②④⑤⑥
Lin et al. (2022)	Lung	LLC, 2 × 10^6^	C57BL/6, M, 18–22g	10,10	APS + anti-PD-L1	80 mg/kg, p.o.,qd ×14	200 ug/mouse, i.p., q4d ×4	7 days post-modeling	14	②④⑤⑥
Li et al. (2024)	Melanoma	B16, 1 × 10^5^	C57BL/6N,M, 6–8 w,18–22g	5,5	APS-NVs	6.25 mg/kg, i.v., q4d ×3	25 mg/kg, i.v., q4d ×3	3 days post-modeling	16	①②⑤⑥⑩⑪
	Lung	LLC, 1 × 10^5^	C57BL/6N,M, 6–8 w,18–22g	6,6	APS-NVs@L-Ag	i.v., q4d ×3	i.v., q4d ×3	3 days post-modeling	16	①②⑩⑪
Cao et al. (2024)	Colon	MC38, NR	C57BL/6, F, 6–8 w	4,4	NP-TCL@APS	100 ul/mouse, s.c.,qw ×3	100 ul/mouse, s.c.,qw ×3	5 days post-modeling	28	①②⑥
Lv et al. (2017)	Liver	H22, 4 × 10^5^	KM mice,M, 6 w,15–25g	10,10	APS + RT	200 mg/kg, i.p., qd ×14	5 Gy qod ×7	7 days post-modeling	14	①③④
Zhang (2019)	Lung	LLC, 2 × 10^6^	C57BL/6J,M, 6–8 w,18–22g	15,15	APS + RT	174 mg/kg, p.o., qd ×7	8Gy qd ×1	9–12 days post-modeling	13	①⑤⑥⑮
Mu et al. (2019)	Liver	H22, 1 × 10^6^	KM mice, M, 8 w	10,10	APS + RT	100 mg/kg, i.p., qd ×10	4Gy qd ×1	When tumor volume reached 100 mm^3^	10	①②④⑤⑮
Song et al. (2022)	Cervical	U14, 1 × 10^6^	KM mice, F, 18–22g	10,10	APS + RT	2 mg/mouse, i.p., qd ×10	4Gy qd ×1	When tumor volume reached 100 mm^3^	10	②⑫
Huang et al. (2008)	Liver	H22, 1 × 10^6^	KM mice, F/M, 6–8 w,18–22g	10,10	APS + DDP	200 mg/kg, i.p., qd ×18	2 mg/kg, i.p., q3d ×6	7 days post-modeling	19	①
Tian (2012)	Liver	H22, 5 × 10^6^	KM mice, M, 6–12 w,18–22g	10,10	APS + DOX	200 mg/kg, i.p., qd ×10	1.25 mg/kg, i.p., qod ×3	When tumor volume reached 100 mm^3^	10	①④⑤⑭
Sun et al. (2013)	Sarcoma	S180, 2 × 10^5^	BALB/c, M, 20–22g	10,10	APS + DDP	300 mg/kg, p.o., qd ×14	2 mg/kg, i.p., qod ×7	2 days post-modeling	14	①④⑩
Ming et al. (2014)	Lung	LLC, 2 × 10^6^	C57BL/6, M, 6–8 w,18–22g	10,10	APS + DDP	100 mg/kg, i.p., qd ×20	6 mg/kg, i.p., qw ×3	2 days post-modeling	21	①④⑤⑫
Zhuang et al. (2017)	Lung	LLC, 2 × 10^6^	C57BL/6J, M, 16–18g	10,10	APS + DDP	60 ug/mouse, i.p., qd ×20	6 mg/kg, i.p., qw ×3	2 days post-modeling	21	①
Wang et al. (2017b)	Lung	LLC, 2 × 10^6^	C57BL/6J, M, 8 w,18–22g	10,10	APS + DDP	200 mg/kg, i.p., qw ×3	2 mg/kg, i.p., qw ×3	When the tumor diameter reached 5 mm	21	①⑥⑮
Wang et al. (2017a)	Breast	EMT-6,1 × 10^6^	BALB/c, NR, 4–5 w	11,11	APS + DOX	100 mg/kg, p.o., q3d ×7	20 mg/kg, i.v., q3d ×7	When tumor volume reached 80 mm^3^	22	②⑥⑩⑪
Zhu et al. (2017)	Glioma	C6, 1 × 10^4^	SD, NR, 6 m,200–220g	15,15	APS + TMZ	250 mg/kg, i.p., qd ×15	20 mg/kg, p.o., qd ×5	Post-modeling	16	②⑤⑮
Zhou et al. (2017b)	NPC	CNE-2,1 × 10^6^	BALB/c-Nu,M, 4-5w,18–20g	5,5	APS + DDP	40 mg/kg,i.p., biw ×8	5 mg/kg, i.p., biw ×8	After successful model establishment	28	①②⑫
Li et al. (2018)	Lung	LLC, 2 × 10^6^	C57BL/6J,F/M 6–8 w,18–20g	10,10	APS + DDP	200 mg/kg, p.o., qd ×21	6 mg/kg, i.p., qw ×3	1 days post-modeling	21	①②⑤⑧⑨⑮
Phacharapiyangkul et al. (2019)	Melanoma	B16F10, NR	C57BL/6, NR	7,7	APS + DDP	50 mg/kg, i.p., q3d ×3	2 mg/kg, i.p., q3d ×3	7 days post-modeling	15	②
	Lung	LLC, NR	C57BL/6, NR	7,7	APS + DDP	50 mg/kg, i.p., q3d ×3	2 mg/kg, i.p., q3d ×3	7 days post-modeling	15	②
Bamodu et al. (2019)	Lung	LLC, 1.5 × 10^3^	C57BL/6, F, 6-8w,22.7–26.3g	10,10	APS + DDP	3 mg/kg, i.p., q2w ×8	0.5 mg/kg, i.p., qd ×112	When tumor volume reached 200 mm^3^	112	①②⑦
Pan (2020)	Liver	HepG2,2 × 10^8^	BALB/c-Nu, F/M, 6–8 w	7,7	APS + DDP + DOX	200 mg/kg, i.v., qd ×10	2 mg/kg DDP+ 5 mg/kg DOX, i.v., qd ×10	10 days post-modeling	14	①②⑧⑨
Pang (2020)	Breast	4T1, 5 × 10^5^	BALB/c, F	5,5	APS-AuNP + PTX	30 mg/kg, i.v., q3d ×6	6 mg/kg, i.v., q3d ×6	When tumor volume reached 70–80 mm^3^	21	①②⑥⑦
Li et al. (2020)	Breast	4T1, 1.5 × 10^6^	BALB/c, F, 6–8 w	15,15	APS+5-FU	200 mg/kg, i.p., qd ×14	20 mg/kg, i.p., qd ×14	When tumor volume reached 100 mm^3^	15	①②④⑤⑫
Wang (2021)	Colon	CT-26,2 × 10^6^	BALB/c, M, 4–6 w,16–20g	5,5	APS+5-FU	200 mg/mL, i.p., q.d.×7	10 mg/mL, i.p., qd ×7	7 days post-modeling	7	①⑮
Zhang et al. (2022c)	Lung	A548/DDP, NR	BALB/c-Nu, M 6 w,18–22g	5,5	APS + DDP	300 mg/kg.p.o.,qd ×24	3.5 mg/kg, i.p., biw ×6	8 days post-modeling	32	①⑦⑭⑮
Qu (2022)	Lung	LLC, 2 × 10^6^	KM mice, F, 6–8 w,18–22g	10,10	APS + DDP	100 mg/kg, i.p., qd ×14	20 mg/kg, i.p., qd ×3	7 days post-modeling	14	①④⑤⑮
Gong et al. (2022)	Melanoma	B16/DDP, 1 × 10^6^	C57BL/6, M, 4–5 w	6,6	APS + DDP	200 mg/kg, i.v., qd ×7	2.5 mg/kg, i.p., q3d ×2	1 day post-modeling	14	①②⑬⑮
Wang (2023)	Colon	CT-26,1 × 10^6^	BALB/c, F, 4–6 w,16–20g	10,10	APS+5-FU	200 mg/kg, i.p., qd ×7	20 mg/kg, i.p., qd ×7	7 days post-modeling	14	②④⑥⑬
Li et al. (2023)	Liver	Hep3B,2 × 10^6^	BALB/c-Nu,M, 4–6 w	5,5	APS + DOX	50 mg/kg, i.p., q3d ×7	2 mg/kg, i.p., q3d ×7	7 days post-modeling	28	①②⑭
Chen (2024)	Bladder	UM-UC-3, 1 × 10^7^	BALB/c-Nu, F, 4 w	4,4	APS + DDP	100 mg/kg, i.p., qd ×21	2 mg/kg, i.p., qw ×4	7 days post-modeling	28	①
Qiu et al. (2024)	Pancreas	PANC-1, NR	SD-Nude, M, 140–160g	10,10	APS + GEM	200 ul/mouse, p.o., qd ×21	100 mg/kg, i.p., qw ×2	After successful model establishment	21	①②⑫
Ma (2024)	Lung	LLC, 2 × 10^6^	BALB/c, F, 4–5 w,16–20g	10,10	APS + DDP	200 mg/kg, p.o., qd ×21	2 mg/kg, i.p., qod ×7	Within 24 h post-modeling	21	①④⑤
Sun et al. (2025)	Breast	4T1, 1 × 10^4^	BALB/c, F, 6 w,16–18g	8,8	APS + DDP	100 mg/kg, i.p., qod ×5	3 mg/kg, i.p., q4d ×3	8 days post-modeling	18	②⑥⑫
Chen et al. (2025)	Cervical	Hela, 1 × 10^6^	BALB/c-Nu, F	7,7	APS + DDP	200 mg/kg, i.p., qd ×14	5 mg/kg, i.p., biw ×4	After successful model establishment	14	①②⑫⑮
Shi et al. (2025)	Lung	LLC, 1 × 10^6^	BALB/c, M, 5 w,18–20g	15,15	APS + DDP	400 mg/kg, i.p., qd ×14	6 mg/kg, i.p., qw ×2	24 h post-modeling	14	④⑤⑦

NPC, nasopharyngeal carcinoma; NR, no report; iv., intravenous injection; i. p., intraperitoneal injection; p. o., oral gavage; s. c., subcutaneous injection; M, male; F, female; w, weeks; qd, once daily; qod, every other day; q3d, every 3 days; q4d, every 4 days; qw, once weekly; biw, twice a week; q2w, every 2 weeks; APS, astragalus polysaccharide; RT, radiotherapy; DDP, cisplatin; TMZ, temozolomide; DOX, doxorubicin; PTX, paclitaxel; 5-FU, 5-Fluorouracil; GEM, gemcitabine; ① Tumor weight; ② Tumor volume; ③ Survival time; ④ Immune organ index; ⑤ Immune cytokines; ⑥ Immune cells; ⑦ Lung metastatic nodules; ⑧ Peripheral blood cells; ⑨ Bone marrow cells; ⑩ Liver function; ⑪ Kidney function; ⑫ Apoptosis-related molecules; ⑬ Immune checkpoint molecules; ⑭ Drug resistance-related molecules; ⑮ Others.

A total of 748 animals were evaluated across eight rodent models, including BALB/c mice (n = 11 studies), C57BL/6 mice (n = 11), KM mice (n = 6), BALB/c nude mice (n = 6), C57BL/6J mice (n = 4), C57BL/6N mice (n = 2), NOD/SCID mice (n = 2), and Sprague-Dawley (SD) rats (n = 2). Group sample sizes ranged from 4 to 15 animals. Male-only models constituted 47% (n = 21) of studies, female-only 30% (n = 13), and mixed-sex models 14% (n = 6), while 9% (n = 4) did not report sex.

APS was primarily investigated as an adjunct to chemotherapy (n = 28, 63.6%), immunotherapy (n = 12, 27.3%; including immune checkpoint inhibitors [n = 5], adoptive cell therapy [n = 4], and cancer vaccines [n = 3]), and radiotherapy (n = 4, 9.1%). Administration routes included intraperitoneal injection (i.p., n = 25, 56.8%), oral gavage (p.o., n = 12, 27.3%), intravenous infusion (i.v., n = 6, 13.6%), and subcutaneous injection (s.c., n = 1, 2.3%). APS sources were categorized into commercial research-grade preparations (n = 28, 63.6%), clinically formulated injections (n = 10, 22.7%), and laboratory-extracted preparations (n = 6, 13.6%). APS dosages ranged from 3 to 400 mg/kg/day, administered over 3–24 days.

### 3.3 Quality assessment of the included studies

**FIGURE 2:**
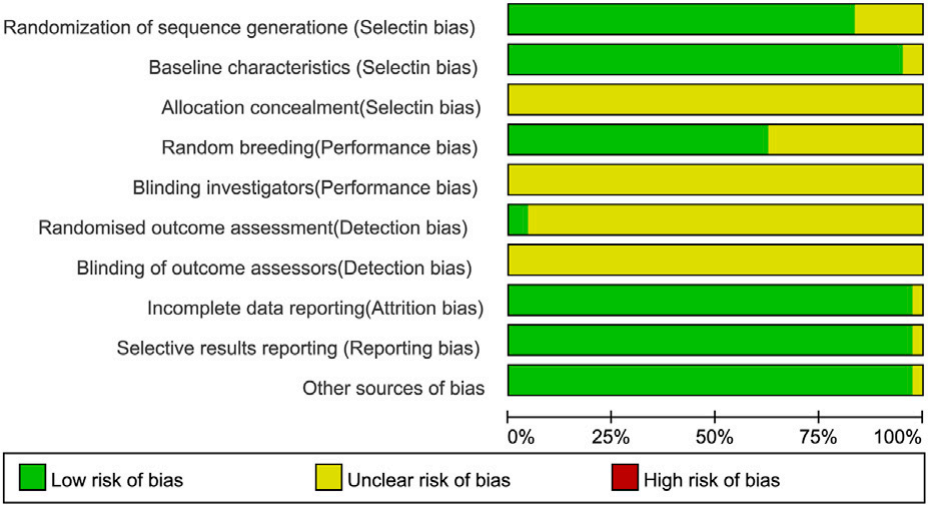
Risk-of-bias graph of the included studies assessed using SYRCLE’s tool for animal experiments.

The risk of bias across all included studies was evaluated using the SYRCLE Risk of Bias tool, as summarized in Figure 2; Supplementary Table S1. For selection bias, random sequence generation was rated low risk in 4 studies that explicitly used random number tables and unclear risk in 7 studies that only implied randomization, while 33 studies merely reported “random allocation” without providing methodological details. Allocation concealment was judged unclear risk in all 44 studies, as none described concealment procedures. Baseline characteristics were considered low risk in 42 studies that reported comparable groups, whereas 2 studies were rated unclear risk due to insufficient baseline information. For performance bias, 28 studies were judged low risk for reporting standardized housing conditions, while 16 studies were rated unclear risk because housing details were inadequately documented. Detection bias was rated low risk in only 2 studies that employed randomized outcome assessment, whereas 42 studies were considered unclear risk owing to unreported assessment procedures. Moreover, none of the 44 studies reported blinding of investigators or outcome assessors. Attrition bias was deemed low risk in all studies, as outcome data were complete. Reporting bias was also judged low risk, with no evidence of selective outcome reporting. No additional sources of bias were identified following a systematic assessment of potential confounding factors.

### 3.4 Outcomes and effectiveness

#### 3.4.1 Tumor weight

**FIGURE 3:**
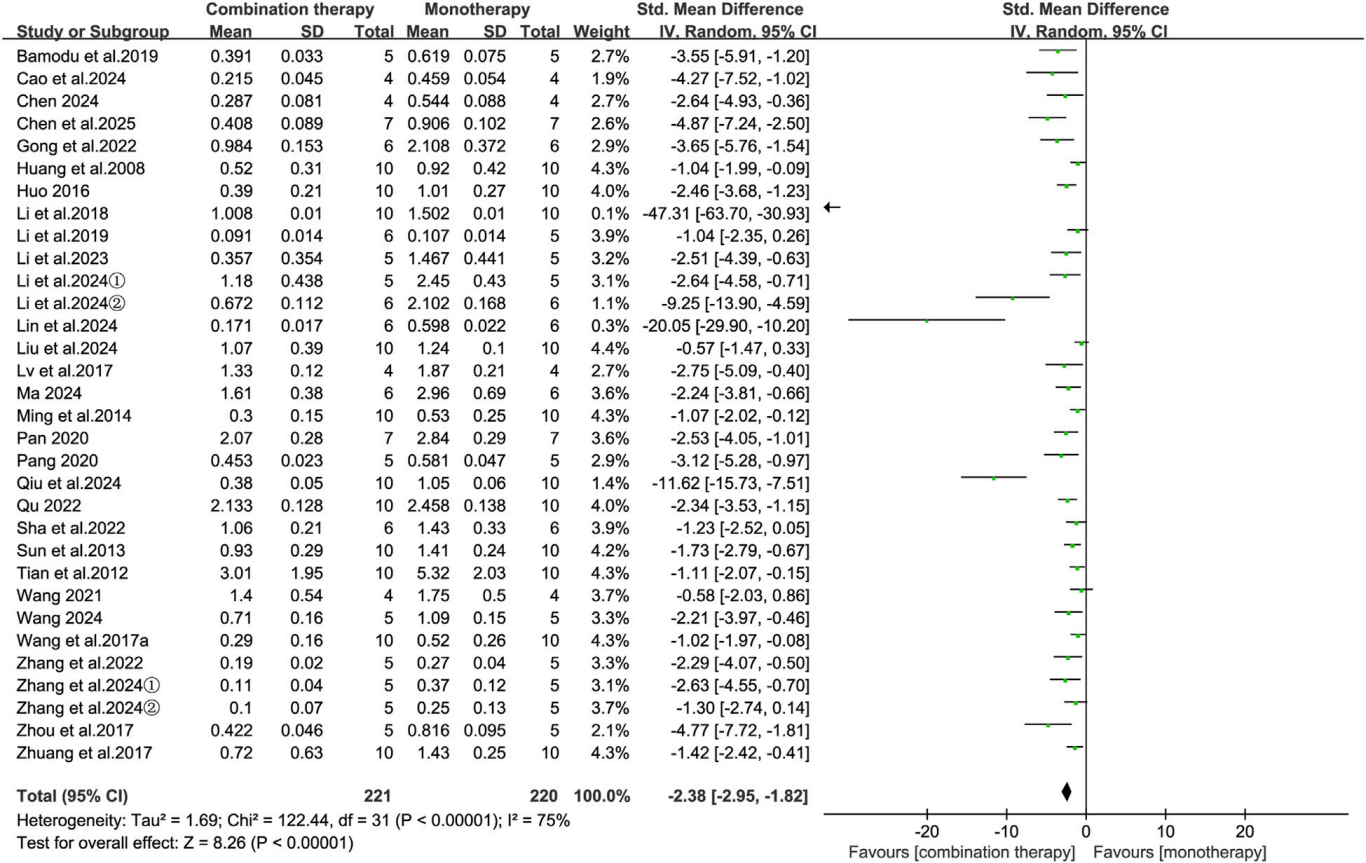
Forest plot of tumor weight reduction comparing the APS combination therapy with monotherapy.

Tumor weight, a primary endpoint in preclinical oncology models, may provide a direct and quantitative indicator of antitumor efficacy. Meta-analysis of 30 publications (32 independent studies) demonstrated that APS combination therapy was associated with a significant reduction in tumor weight compared with conventional monotherapy (SMD = −2.38, 95% CI [-2.95, −1.82], *p* < 0.00001; Figure 3). Given substantial heterogeneity (I^2^ = 75%; *p* < 0.001), we conducted meta-regression and subgroup analyses to explore potential sources. Under the random-effects model, meta-regression identified no significant associations between heterogeneity and any prespecified covariates—including species, model type, cancer type, treatment regimen, APS source, treatment initiation timing, treatment duration, or administration route (all *p* ≥ 0.05; Supplementary Table S2).

However, subgroup analyses identified model type (immunocompetent vs. immunodeficient; I^2^ = 70.2%, *p* = 0.07), administration route (I^2^ = 80.7%, *p* = 0.006), and treatment initiation timing (I^2^ = 60.2%, *p* = 0.04) as significant contributors to heterogeneity (Table 2). In contrast with the overall results, subgroup analyses by cancer type showed no statistically significant reduction in tumor weight in breast cancer models (SMD = −1.90, 95% CI [-3.91, 0.10], *p* = 0.06) or colon cancer models (SMD = −2.13, 95% CI [-5.69, 1.43], *p* = 0.24), indicating that the therapeutic advantage of APS may not evident in these two cancer types. Notably, the reduction in tumor weight was greater when APS was combined with immunotherapy (SMD = −2.71, 95% CI [-3.89, −1.53], *p* < 0.00001) compared to chemotherapy (SMD = −2.35, 95% CI [-3.04, −1.66], *p* < 0.00001).

**TABLE 2:** Subgroup analysis for tumor weight.

Subgroup	Participants			Test for heterogeneity		Test for effect		Test for subgroup difference	
	Studies	Com	Mon	*I* ^2^ (%)	*P*-value	SMD [95% CI]	*P*-value	*I* ^2^ (%)	*P*-value
Species									
BALB/c	13	80	79	60	0.0003	−2.45 [-3.23, −1.67]	<0.0001	3.0	0.38
C57BL/6	12	87	87	82	<0.00001	−2.23 [-3.31, −1.16]	<0.0001		
KM mice	4	34	34	34	0.21	−1.54 [-2.27, −0.80]	<0.0001		
NOD/SCID	2	10	10	14	0.28	−1.80 [-3.06, −0.54]	0.005		
SD	1	10	10	-	-	-	-		
Model type									
Immunocompetent	23	168	167	74	<0.00001	−2.03 [-2.64, −1.42]	<0.00001	70.2	0.07
Immunodeficient	9	53	53	70	0.0007	−3.34 [-4.60, −2.08]	<0.00001		
Tumor type									
Lung cancer	11	87	87	81	<0.00001	−2.19 [-3.22, −1.17]	<0.0001	0	0.73
Liver cancer	8	52	52	66	0.005	−2.01 [-2.99, −1.04]	<0.0001		
Melanoma	3	17	17	51	0.13	−2.31 [-3.76, −0.87]	0.002		
Breast cancer	2	11	10	62	0.11	−1.90 [-3.91, 0.10]	0.06		
Cervical cancer	2	17	17	68	0.08	−3.44 [-5.77, −1.11]	0.004		
Colon cancer	2	8	8	76	0.04	−2.13 [-5.69, 1.43]	0.24		
Others	4	29	29	87	<0.0001	−4.71 [-7.95, −1.47]	0.004		
Combined therapy									
Immunotherapy	10	62	62	75	<0.00001	−2.71 [-3.89, −1.53]	<0.00001	0	0.61
Chemotherapy	21	155	154	77	<0.00001	−2.35 [-3.04, −1.66]	<0.00001		
Radiotherapy	1	4	4	-	-	-	-		
APS Source									
Clinical-grade Injection	6	48	47	56	0.05	−1.73 [-2.52, −0.95]	<0.0001	31.6	0.23
Research grade	21	141	141	79	<0.00001	−2.71 [-3.54, −1.88]	<0.00001		
Self-extracted	5	32	32	73	0.005	−2.46 [-3.97, −0.94]	0.001		
Administration route									
i.p	16	115	115	50	0.01	−1.63 [-2.11, −1.14]	<0.00001	80.7	0.006
i.v	6	39	39	41	0.13	−3.08 [-4.10, −2.06]	<0.00001		
p.o	9	63	63	88	<0.00001	−3.93 [-5.86, −2.00]	<0.0001		
s.c	1	4	4	-	-	-	-		
Treatment initiation									
1–6 days post-modeling	13	92	92	76	<0.00001	−2.54 [-3.49, −1.59]	<0.00001	64.2	0.04
7–12 days post-modeling	10	64	64	45	0.06	−1.77 [-2.41, −1.14]	<0.00001		
When tumor volume reached 50–200 mm^3^	6	43	42	72	0.003	−1.65 [-2.80, −0.51]	0.005		
NR	3	22	22	75	<0.00001	−6.76 [-10.47,-3.05]	0.0004		
Duration									
≤14 days	12	85	85	70	0.0001	−2.04 [-2.84, −1.25]	<0.00001	11.4	0.29
>14 days	20	136	135	78	<0.00001	−2.66 [-3.46, −1.85]	<0.00001		

#### 3.4.2 Tumor volume

**FIGURE 4:**
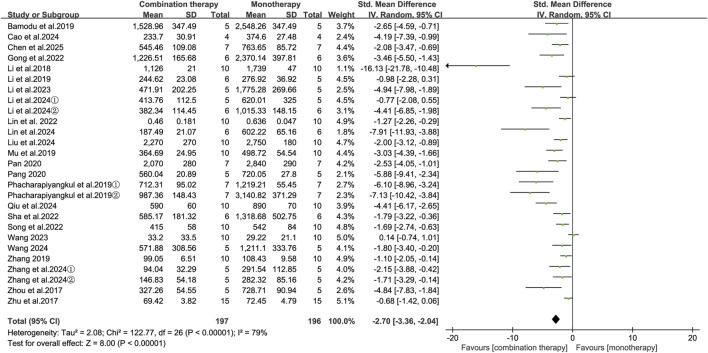
Forest plot of tumor volume reduction comparing the APS combination therapy with monotherapy.

Tumor volume is commonly used as another primary endpoint for assessing antitumor efficacy. Analysis of 24 articles (27 studies) revealed that combined APS and antitumor therapy was associated with a significant reduction in tumor volume compared to monotherapy (SMD = −2.70, 95% CI [-3.36, −2.04], *p* < 0.00001; Figure 4). Substantial heterogeneity was observed across the studies (I^2^ = 79%, *p* < 0.00001). Meta-regression of prespecified covariates identified no significant heterogeneity sources (all *p* ≥ 0.05; Supplementary Table S2). Subgroup analyses suggested that the combination regimen (*I* ^2^ = 60.1%, *p* = 0.08) and treatment duration (*I* ^2^ = 84.4%, *p* = 0.01) were significant contributors to heterogeneity. Similarly, in breast cancer models (SMD = −3.14, 95% CI [-7.91, 1.62], *p* = 0.20) and colon cancer models (SMD = −1.74, 95% CI [-5.94, 2.46], *p* = 0.42), APS combination therapy did not produce a statistically significant reduction in tumor volume compared with monotherapy controls (Table 3).

**TABLE 3:** Subgroup analysis for tumor volume.

Subgroup	Participants			Test for heterogeneity		Test for effect		Test for subgroup difference	
	Studies	Com	Mon	I2 (%)	P-value	SMD [95% CI]	P-value	I2 (%)	P-value
Species									
BALB/c	8	51	51	83	<0.00001	−3.03 [-4.60, −1.46]	0.0002	0	0.65
C57BL/6	13	91	91	79	<0.0001	−3.03 [-4.07, −1.98]	<0.00001		
KM mice	2	20	20	57	0.13	−2.28 [-3.59, −0.98]	0.006		
NOD/SCID	2	10	10	0	0.72	−1.91 [-3.07, −0.75]	0.001		
SD rat	2	25	25	93	0.0001	−2.46 [-6.10, 1.19]	0.19		
Model type									
Immunocompetent	20	153	152	81	<0.0001	−2.62 [-3.40, −1.83]	<0.00001	0	0.68
Immunodeficient	7	44	44	42	0.11	−2.87 [-3.79, −1.96]	<0.00001		
Tumor type									
Lung cancer	7	56	56	82	<0.0001	−2.69 [-4.04, −1.34]	<0.0001	0	0.52
Liver cancer	7	45	45	65	0.009	−3.51 [-4.82, −2.19]	<0.0001		
Melanoma	4	24	24	78	0.004	−2.71 [-4.60, −0.83]	0.005		
Breast cancer	2	11	10	85	0.01	−3.14 [-7.91, 1.62]	0.20		
Cervical cancer	2	17	17	0	0.65	−1.83 [-2.67, −0.99]	<0.0001		
Colon cancer	2	14	14	85	0.01	−1.74 [-5.94, 2.46]	0.42		
Others	3	30	30	90	<0.00001	−3.12 [-6.19, −0.06]	0.05		
Combined therapy									
Immunotherapy	10	62	62	53	0.02	−2.10 [-2.85, −1.35]	<0.00001	60.1	0.08
Radiotherapy	3	30	30	61	0.08	−1.84 [-2.87, −0.81]	0.0005		
Chemotherapy	14	105	104	87	<0.00001	−3.59 [-4.85, −2.32]	<0.00001		
APS Source									
Clinical-grade Injection	8	67	66	79	<0.0001	−2.64 [-3.78, −1.50]	<0.00001	0	0.97
Research grade	15	108	108	82	<0.00001	−2.83 [-3.82, −1.85]	<0.00001		
Self-extracted	4	22	22	75	0.008	−2.74 [-4.67, −0.81]	0.005		
Administration route									
i.p	13	102	101	79	<0.00001	−2.36 [-3.24, −1.49]	<0.00001	0	0.59
i.v	5	29	29	70	0.01	−3.03 [-4.64, −1.41]	0.0002		
p.o	8	62	62	85	<0.00001	−3.14 [-4.61, −1.68]	<0.0001		
s.c	1	4	4	-	-	-	-		
Treatment initiation									
1–6 days post-modeling	9	61	61	82	<0.00001	−2.78 [-4.14, −1.43]	<0.0001	0	0.72
7–12 days post-modeling	9	71	71	84	<0.00001	−2.76 [-4.00, −1.52]	<0.0001		
When tumor volume reached 50–200 mm^3^	6	43	42	63	0.02	−2.31 [-3.36, −1.25]	<0.0001		
NR	3	22	22	63	0.07	−3.54 [-5.38, −1.70]	0.002		
Duration									
≤14 days	12	97	97	69	0.0002	−1.90 [-2.59, −1.22]	<0.00001	84.4	0.01
>14 days	15	100	99	83	<0.00001	−3.72 [-4.94, −2.49]	<0.00001		

#### 3.4.3 Survival and metastasis outcomes

**FIGURE 5:**
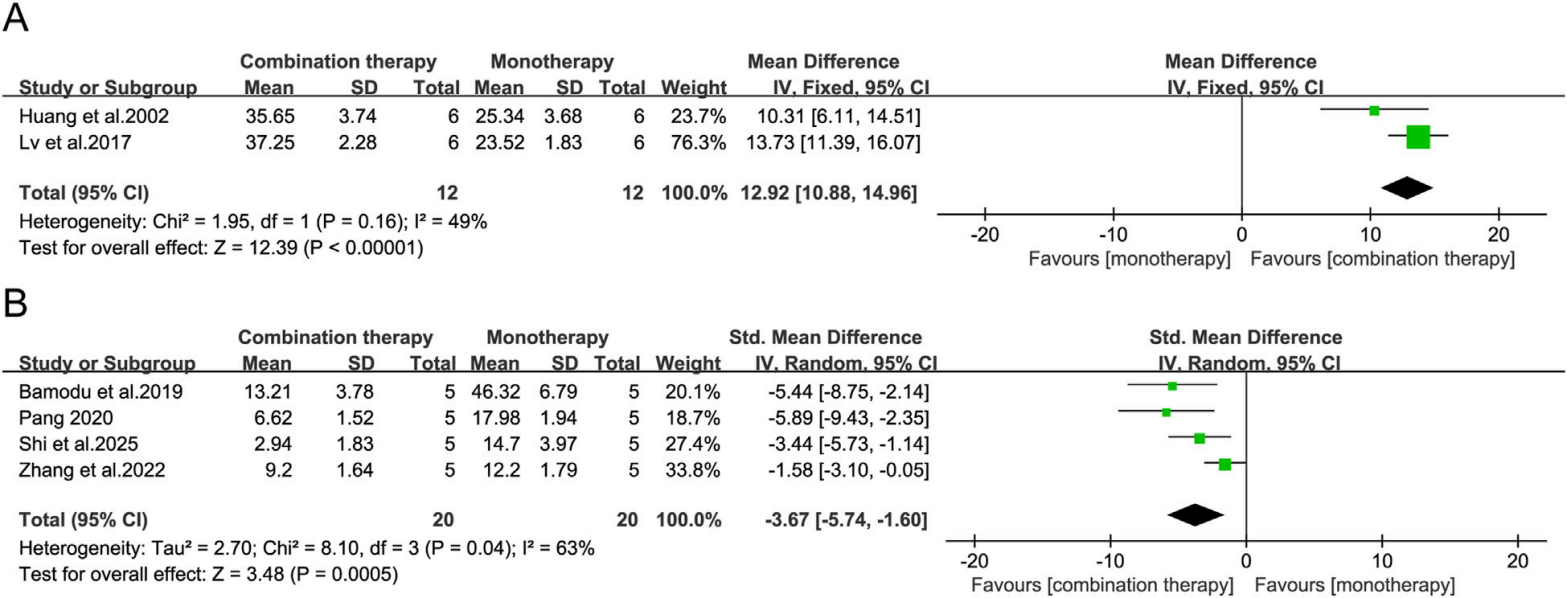
Forest plots of **(A)** survival time and **(B)** lung metastatic nodules in the APS combination therapy vs. monotherapy groups.

Survival time is the most clinically relevant endpoint for evaluating antitumor efficacy. Notably, only 2 of the 44 included studies (4.5%) reported analyzable survival data, substantially limiting the generalizability of these findings. Pooled analysis of these limited studies showed a significant prolongation of median survival time with APS combination therapy compared to monotherapy (MD = 12.92 days, 95% CI [10.88, 14.96], *p* < 0.00001; Figure 5A). Pulmonary metastatic nodule count, an established quantitative measure of metastatic burden and an independent prognostic factor, was assessed as a secondary efficacy endpoint. Pooled data from four studies demonstrated that APS-chemotherapy regimens were associated with a significant reduction in lung metastasis (SMD = −3.67, 95% CI [-5.74, −1.60], *p* = 0.0005; Figure 5B).

#### 3.4.4 Immune organ index

**FIGURE 6:**
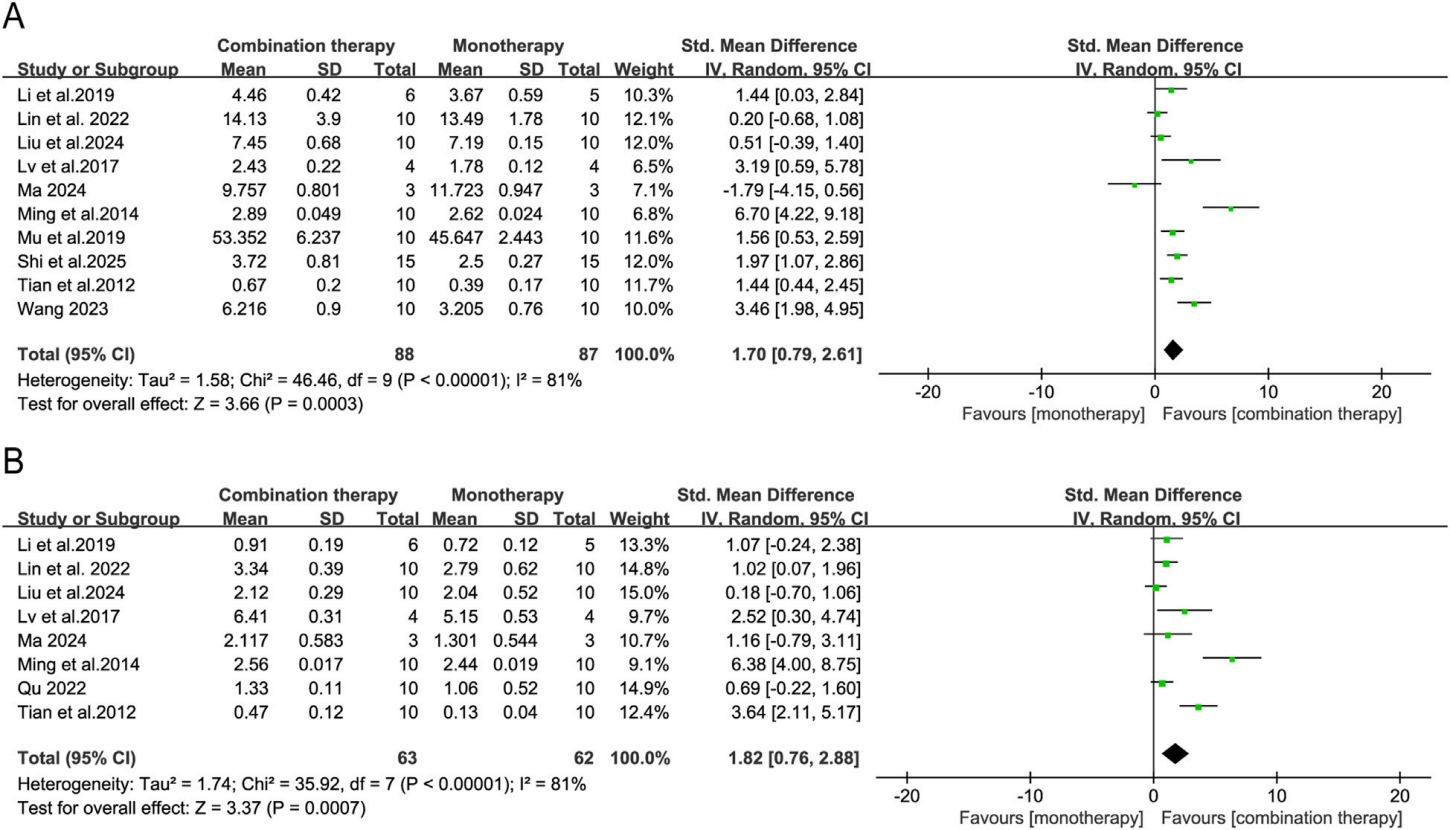
Forest plots of immune organ indices: **(A)** spleen index and **(B)** thymus index in the APS combination therapy vs. monotherapy groups.

The thymus and spleen indices—calculated as organ weight-to-body weight ratios—could serve as quantitative indicators of immunoenhancement or immunosuppression during antitumor therapy. Pooled analyses showed that APS combination therapy significantly increased the spleen index (10 studies; SMD = 1.70, 95% CI [0.79, 2.61], *p* = 0.0003) and thymus index (8 studies; SMD = 1.82, 95% CI [0.76, 2.88], *p* = 0.0007) compared with monotherapy (Figure 6).

#### 3.4.5 CD4^+^ T cells and CD8^+^ T cells

**FIGURE 7:**
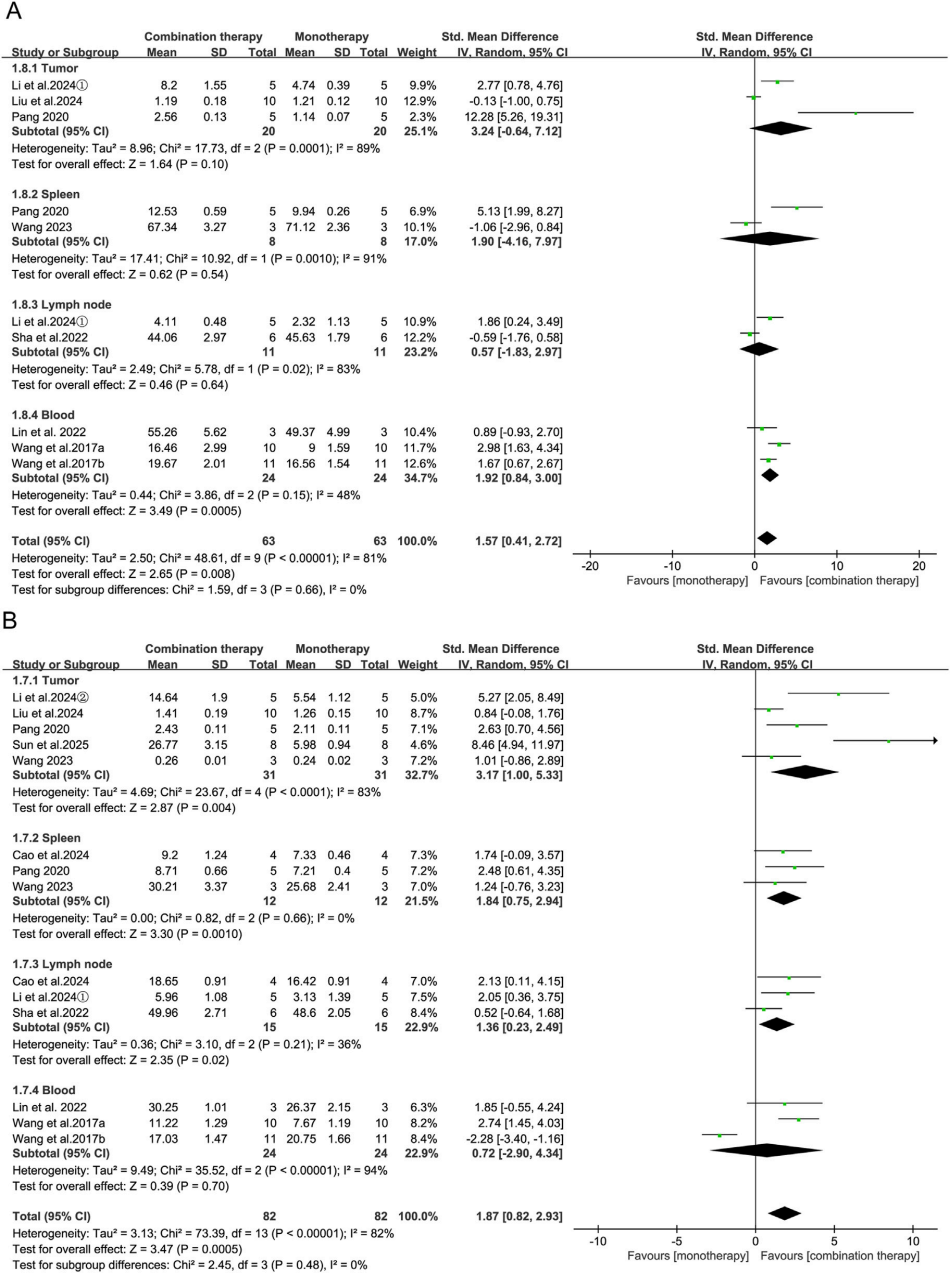
Forest plots of **(A)** CD4^+^ T-cell and **(B)** CD8^+^ T-cell levels comparing the APS combination therapy with monotherapy.

Increased CD8^+^ T-cell infiltration in the tumor, spleen, and draining lymph nodes generally reflects enhanced systemic antitumor immune activation. Meta-analysis of 14 studies showed that APS combination therapy significantly increased CD8^+^ T-cell proportions in tumor tissue (SMD = 3.17, 95% CI [1.00, 5.33], *p* = 0.004), spleen (SMD = 1.84, 95% CI [0.75, 2.94], *p* = 0.001) and draining lymph nodes (SMD = 1.36, 95% CI [0.23, 2.49], *p* = 0.02), respectively. Notably, peripheral blood CD8^+^ T-cell frequencies remained unchanged (SMD = 0.72, 95% CI [−2.90, 4.34], *p* = 0.70). In contrast, pooled analyses of 10 studies revealed that APS combination therapy significantly elevated peripheral blood CD4^+^ T-cell frequencies (SMD = 1.92, 95% CI [0.84, 3.00], *p* = 0.0005), while tumor-infiltrating and lymphoid tissue-resident CD4^+^ T-cell populations showed no significant alterations (all *p* > 0.05; Figure 7). This compartmentalized distribution pattern may reflect distinct trafficking dynamics between CD4^+^ and CD8^+^ T-cell subsets in response to APS-enhanced immunotherapy.

#### 3.4.6 Cytokine levels

**FIGURE 8:**
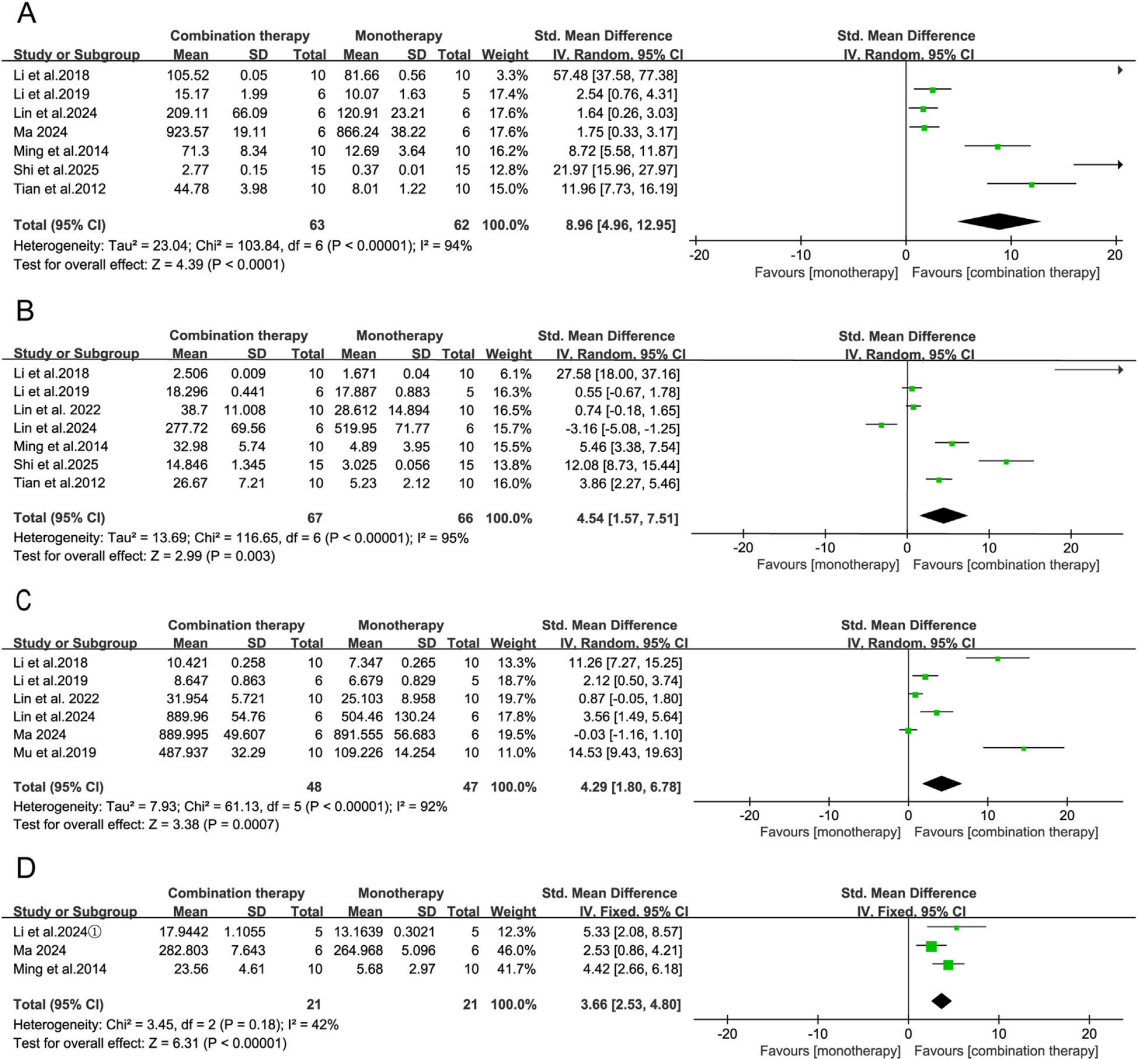
Forest plots of serum cytokine levels: **(A)** TNF-α, **(B)** IL-2, **(C)** IFN-γ, and **(D)** IL-12 in the APS combination therapy vs. monotherapy groups.

**FIGURE 9:**
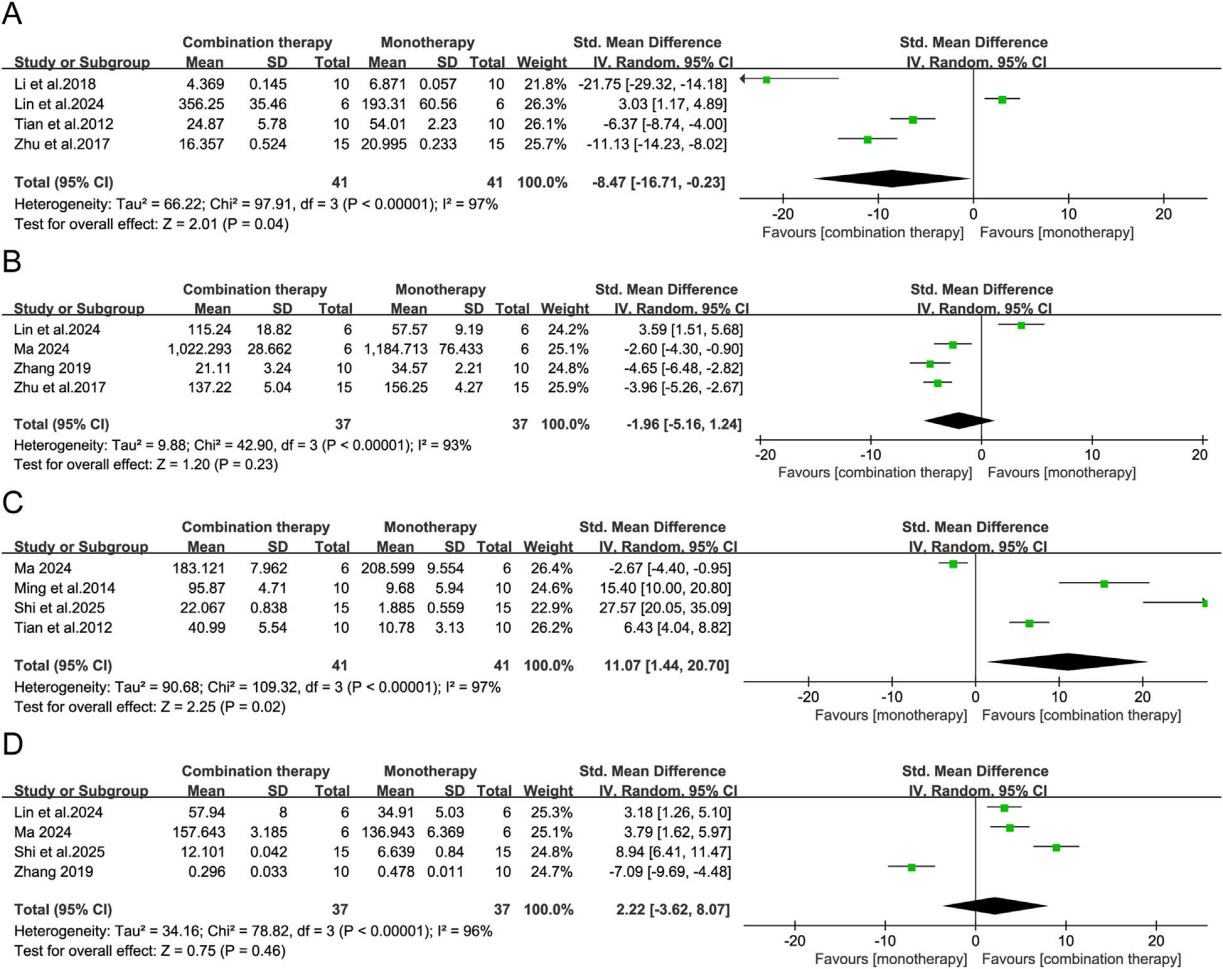
Forest plots of serum cytokine levels: **(A)** IL-10, **(B)** TGF-β, **(C)** IL-6, and **(D)** IL-1β in the APS combination therapy vs. monotherapy groups.

As pivotal immune regulators, cytokines orchestrate antitumor responses both within the tumor microenvironment and systemically. In the included preclinical studies, APS combination therapy was reported to alter several serum cytokines, although interpretation is constrained by notable methodological limitations and substantial between-study heterogeneity. Pooled analyses indicated higher concentrations of tumor necrosis factor-α (TNF-α: SMD = 8.96, 95% CI [4.96, 12.95], *p* < 0.0001), interleukin-2 (IL-2: SMD = 4.54, 95% CI [1.57, 7.51], *p* = 0.003), interferon-γ (IFN-γ: SMD = 4.29, 95% CI [1.80, 6.78], *p* = 0.0007), interleukin-12 (IL-12: SMD = 3.66, 95% CI [2.53, 4.80], *p* < 0.00001), and interleukin-6 (IL-6: SMD = 11.07, 95% CI [1.44, 20.70], *p* = 0.02) in the APS combination groups. A reduction in interleukin-10 was also observed (IL-10: SMD = −8.47, 95% CI [-16.71, −0.23], *p* = 0.04). No statistically significant differences were detected for transforming growth factor-β (TGF-β: SMD = −1.96, 95% CI [-5.16, 1.24], *p* = 0.23) or interleukin-1β (IL-1β: SMD = 2.22, 95% CI [-3.62, 8.07], *p* = 0.46). (Figures 8, 9).

#### 3.4.7 Immune checkpoint

**FIGURE 10:**
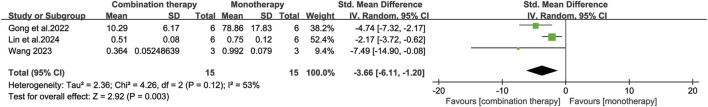
Forest plot of PD-1/PD-L1 expression in tumor tissues comparing the APS combination therapy with monotherapy.

Programmed cell death protein 1 (PD-1) and its ligand PD-L1 represent critical immune checkpoint molecules that mediate tumor immune evasion. Among the included studies, three provided evaluable PD-1/PD-L1 expression data. Meta-analysis revealed that APS combination therapy significantly downregulated PD-1/PD-L1 expression in tumor tissues (SMD = −3.66, 95% CI [-6.11, −1.20], *p* = 0.003; Figure 10).

#### 3.4.8 Safety assessment

The safety profile of APS combination therapy was evaluated in eight included studies, which assessed general animal condition, hepatic and renal function parameters, and organ-specific histopathology. Of these, four studies (Huang et al., 2008; Tian, 2012; Ma, 2024; Chen et al., 2025) assessing general health status—such as daily activity, mental state, and coat appearance—reported no APS-related adverse effects. Notably, two of these studies indicated that mice in the combination therapy groups exhibited better mental status compared to those receiving chemotherapy alone. Histological evaluations of organs in three studies (Wang et al., 2017b; Ma, 2024; Sun et al., 2025) showed no signs of drug-induced tissue injury.

**FIGURE 11:**
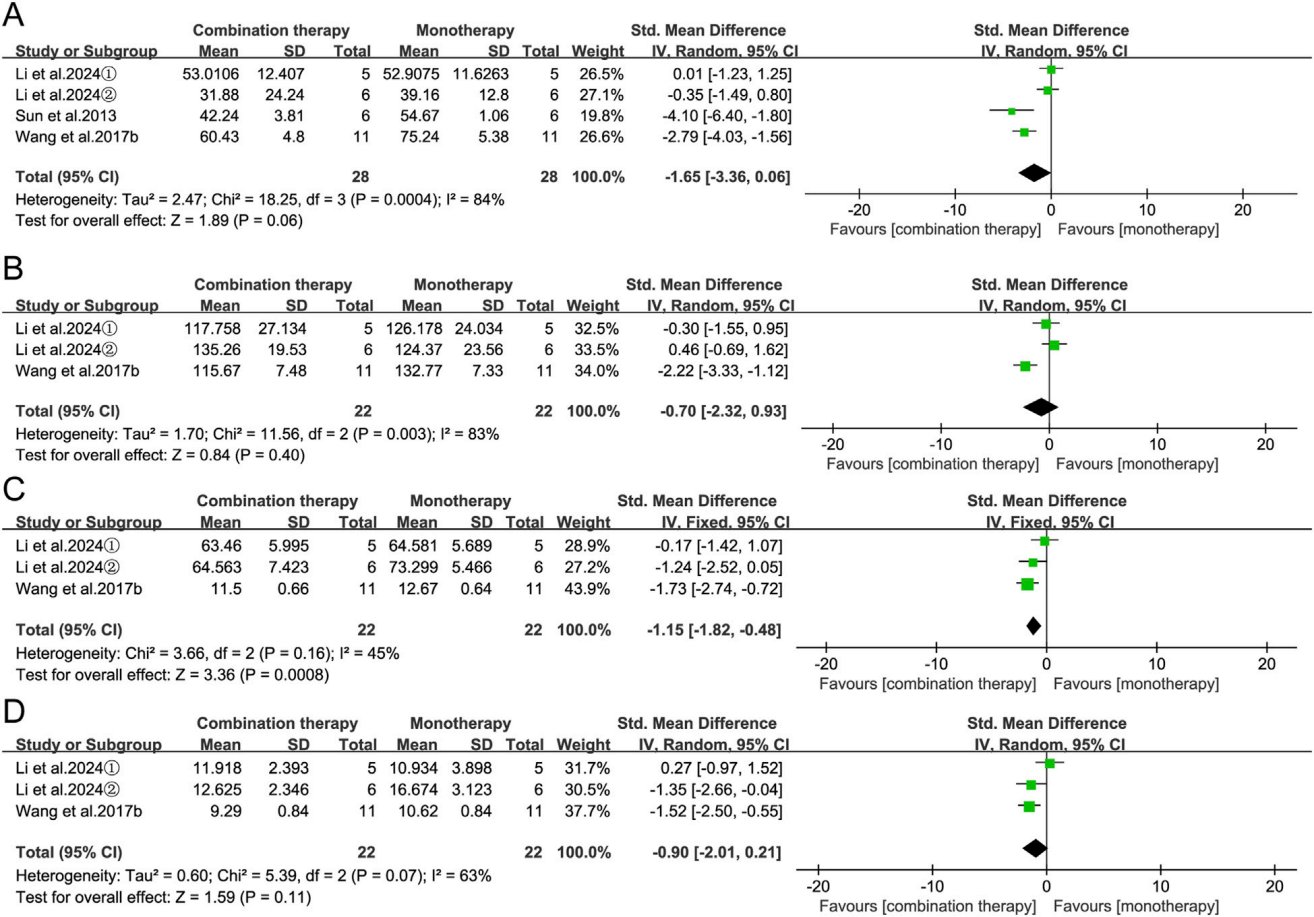
Forest plots of organ function biomarkers: **(A)** ALT, **(B)** AST, **(C)** CRE, and **(D)** UBN in the APS combination therapy vs. monotherapy groups.

Based on data availability, serum creatinine (CRE), blood urea nitrogen (BUN), aspartate aminotransferase (AST), and alanine aminotransferase (ALT) were incorporated into the meta-analysis. The result showed that APS combination therapy was associated with improved renal function, evidenced by decreased CRE levels (SMD = −1.15, 95% CI [-1.82, −0.48], *p* = 0.0008). A trend toward hepatoprotection was also observed, with ALT levels approaching statistical significance (SMD = −1.65, 95% CI [-3.36, 0.06], *p* = 0.06), whereas AST and BUN levels did not change significantly (Figure 11).

In addition, two studies evaluated hematological outcomes. Both studies consistently reported that APS combined with chemotherapy led to higher counts of bone marrow cells, platelets (PLT), white blood cells (WBC), and red blood cells (RBC) relative to chemotherapy alone, suggesting a potential hematopoietic-supportive effect of APS.

### 3.5 Sensitivity analysis

Sensitivity analysis confirmed the robustness of the primary outcomes—including tumor weight and volume, survival time, metastatic nodules, immune organ indices (thymus and spleen), T-cell subset frequencies (CD4^+^/CD8^+^), and key cytokines (IL-12, IFN-γ, TNF-α, IL-10, and IL-2)—as the iterative exclusion of individual studies did not materially affect pooled effect sizes or heterogeneity patterns. Four outcomes exhibited sensitivity to individual study exclusion. Specifically, PD-1/PD-L1 effects lost statistical significance (*p* > 0.05) upon removal of Gong et al. (2022); IL-6 lost significance (*p* > 0.05) when any of three studies were omitted (Ming et al., 2014; Tian, 2012; Shi et al., 2025); IL-10 similarly lost significance when excluding Zhu et al. (2017), Tian (2012), or Li et al. (2018); conversely, IL-1β became statistically significant (*p* < 0.05) after exclusion of Zhang (2019).

### 3.6 Publication bias and evidence quality

**FIGURE 12:**
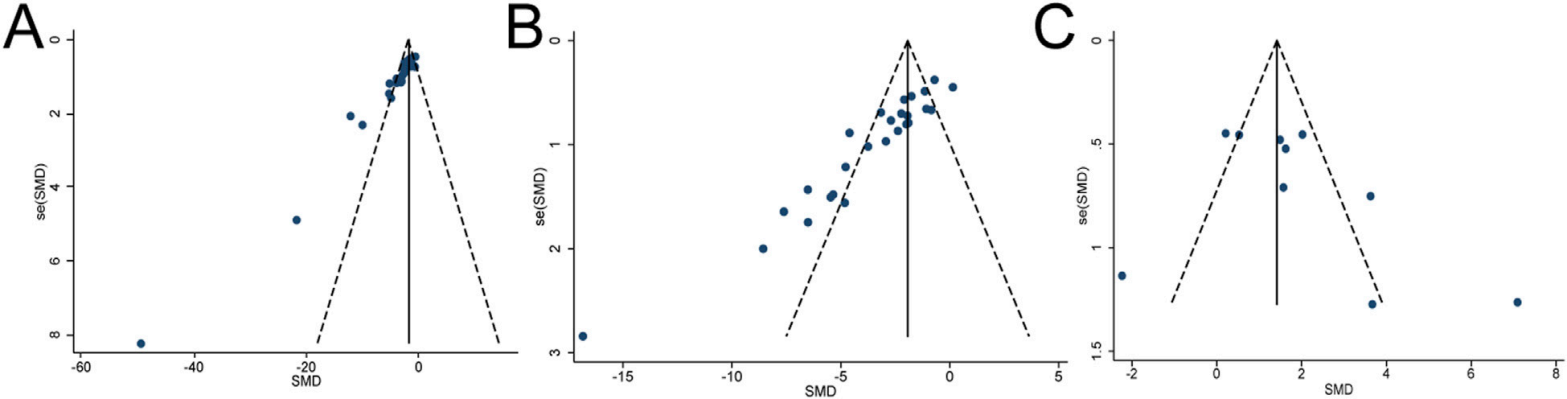
Funnel plots of **(A)** tumor weight, **(B)** tumor volume, and **(C)** spleen index from the included studies.

**FIGURE 13:**
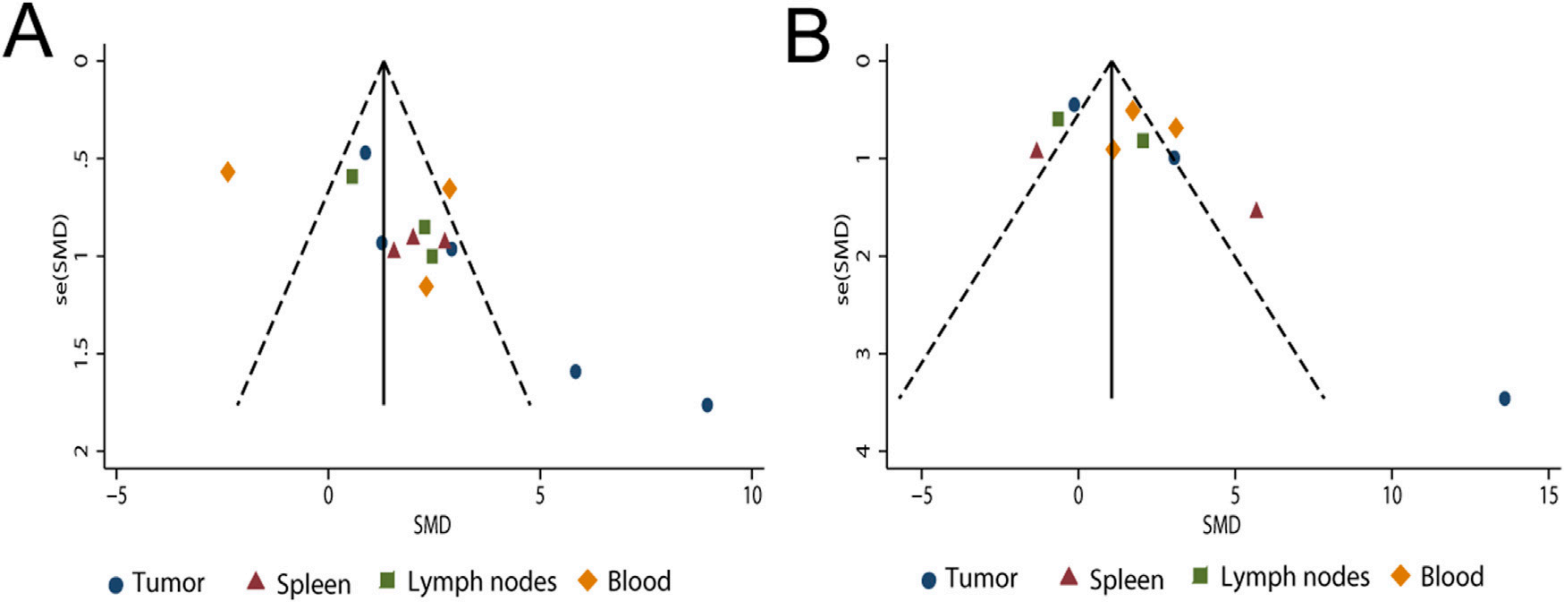
Funnel plots of **(A)** CD8^+^ T cells and **(B)** CD4^+^ T cells from the included studies.

Publication bias assessment for outcomes with ten or more studies revealed no evidence of bias for CD4^+^ T-cell frequencies or the spleen index, as indicated by symmetrical funnel plots and non-significant Egger’s tests (*p* > 0.05). In contrast, significant funnel asymmetry, supported by Egger’s test (*p* < 0.05), suggested potential publication bias for tumor volume, tumor weight, and CD8^+^ T-cell frequencies (Figures 12, 13).

Trim-and-fill analyses indicated that two imputed studies modestly reduced the effect size for tumor weight (adjusted SMD = −0.52, 95% CI [-0.64, −0.40] vs. original SMD = −0.48, 95% CI [-0.60, −0.36], ΔSMD = −0.04), although statistical significance was retained (*p* < 0.001). In contrast, no imputed studies were generated for tumor volume or CD8^+^ T-cell frequencies, and their effect sizes remained unchanged (Supplementary Table S3). According to the GRADE framework, the certainty of evidence for the 21 evaluated outcomes ranged from very low to low. Low-certainty ratings were assigned to six outcomes—thymus index, lung metastatic nodule count, survival time, TNF-α, IFN-γ, and IL-12—while the remaining fifteen outcomes were rated as very low certainty. Detailed GRADE assessments and downgrading domains—including risk of bias, inconsistency, indirectness, imprecision, and publication bias—are provided in Supplementary Table S4.

## 4 Discussion

### 4.1 Summary of the evidence and main findings

Many natural polysaccharides are increasingly recognized as valuable adjuvants in cancer combination therapy (Li et al., 2021; Ornelas et al., 2022). In particular, APS, a bioactive extract from *Astragali Radix*, has attracted considerable attention and is being extensively investigated for its role in potentiating conventional antitumor treatments (Bamodu et al., 2019; Xu et al., 2023; He et al., 2024). Therefore, we herein report the first systematic review and meta-analysis to comprehensively evaluate the efficacy, safety, and mechanistic basis of APS as an adjunctive therapy in animal tumor models.

Our synthesis of 44 preclinical studies (n = 748 animals) indicates that APS co-administration significantly reduced tumor burden, suppressed pulmonary metastasis, and moderately improved survival (all *p* < 0.05), potentially by enhancing T-cell immunity and upregulating antitumor cytokines. A favorable safety profile was also observed. However, according to GRADE assessments, the certainty of evidence for all primary outcomes was low or very low, indicating that these findings should be interpreted cautiously and require validation in rigorously controlled preclinical studies.

### 4.2 Exploration of heterogeneity and its Implications​

Substantial heterogeneity was observed across most primary efficacy outcomes. Meta-regression analyses of eight covariates (e.g., species, model, therapy type) failed to identify significant heterogeneity sources (all *p* ≥ 0.05; Supplementary Table S3). This implies that the heterogeneity likely stems from multiple interacting methodological and biological factors rather than a single dominant contributor.

Subgroup analyses identified administration route as a major contributor to heterogeneity in tumor weight (between-subgroup I^2^ = 80.7%, *p* = 0.006), with oral APS demonstrating superior efficacy compared to parenteral delivery. This pattern may reflect route-dependent differences in pharmacokinetic and immunological engagement. Specifically, oral APS undergoes M cell-mediated transcytosis in Peyer’s patches, activating dendritic cell TLR4 signaling and initiating gut-associated lymphoid tissue (GALT)-primed systemic immunity (Zhang et al., 2022a; 2022b); unabsorbed polysaccharides are subsequently metabolized by colonic microbiota into short-chain fatty acids, further enhancing immunomodulation (Ye et al., 2023). In contrast, parenteral administration bypasses enteric immune activation and relies primarily on direct interaction between circulating APS and systemic immune cells, making its efficacy more dependent on precise structural properties (Cai et al., 2023; Wang et al., 2024). While compelling, these mechanistic explanations remain preliminary due to insufficient reporting of phytochemical and PK/PD data, rendering any definitive conclusion premature. Thus, future studies employing well-characterized APS preparations and detailed PK/PD analyses are essential for validation.

A critical yet unquantified source of between-study variability is the structural heterogeneity of APS itself. The included studies used APS derived from various sources, each subject to distinct extraction and purification procedures that influence molecular weight distribution, branching patterns, and receptor-binding properties (Tang and Huang, 2022). Although subgroup analyses did not detect statistically significant differences among APS sources, this null finding may reflect low statistical power or coarse categorization rather than true equivalence.

Model immune status was identified as a notable contributor to heterogeneity. Subgroup analysis revealed numerically stronger synergistic effects in immunodeficient models (tumor weight: SMD = −3.34; tumor volume: SMD = −2.87) compared to immunocompetent models (tumor weight: SMD = −2.03; tumor volume: SMD = −2.62). This pattern should not be interpreted as superior APS efficacy in the absence of immunity, but may reflect the predominance of immune-independent mechanisms (e.g., direct apoptosis or autophagy induction) combined with reduced biological variability inherent to immunodeficient systems. However, this finding derives from subgroup analysis with limited mechanistic data, warranting cautious interpretation and rigorous experimental validation.

Additional sources of heterogeneity include differences in treatment timing, intervention duration, and the combination therapy. Tumor type-specific responses, such as no significant effects observed in breast and colon cancer models, likely reflect tumor microenvironment responsiveness to APS or low statistical power from an insufficient number of studies. Unmeasured experimental factors—such as tumor inoculation protocols, cell line characteristics, housing conditions, and outcome assessment methods—may further contribute to cross-study inconsistencies.

### 4.3 Potential mechanisms underlying the observed effects

Building upon the subgroup findings, mechanistic indicators extracted from the included studies (Table 4) suggest that APS may influence antitumor responses through both immune-dependent and immune-independent pathways. Immune-mediated mechanisms appear to contribute substantially to APS activity. Increases in spleen and thymus indices in tumor-bearing mice imply that APS co-administration may mitigate lymphoid organ atrophy associated with tumor progression or chemotherapy, thereby preserving the structural basis for immune activation. Enhancements in CD8^+^ T-cell infiltration within tumors and secondary lymphoid organs, together with elevated levels of cytokines such as IFN-γ, TNF-α, IL-2, and IL-12, further indicate partial restoration of antitumor immunity. Concurrent reductions in PD-1/PD-L1 expression and IL-10 levels suggest attenuation of immunosuppressive signaling. Although the mechanistic signals identified in our analysis are constrained by low-quality evidence, similar immune-enhancing effects have been reported in clinical studies of APS injection, including improvements in immune cell subsets (Li and Jiang, 2024) and modulation of inflammatory cytokines (Huang et al., 2019; Mok et al., 2024). This convergence between preclinical and clinical findings provides preliminary support for APS-mediated immunomodulation, although the underlying mechanisms will require further confirmation in rigorously controlled studies.

**TABLE 4:** Outcome measures reported ≥2 times.

Outcome measures	Counts	Outcome measures	Counts
Tumor weight	32	TGF-β levels of blood	3
Tumor volume	27	IL-10 levels of tumor	3
T cells of tumor	11	TGF-β levels of tumor	3
Spleen index	10	TNF-α levels of tumor	3
Thymus index	8	T cells of Lymph node	3
TNF-α levels of blood	7	VEGF	3
IL-2 levels of blood	7	p53 (Western blot)	2
IFN-γ levels of blood	6	p53 mRNA	2
Lung metastatic nodules	4	P-glycoprotein (Western blot)	2
T cells of spleen	4	Liver index	2
T cells of blood	4	Caspase-3 (Western blot)	2
IL-10 levels of blood	4	Bone marrow cells	2
IL-1β levels of blood	4	Platelets (PLT)	2
IL-6 levels of blood	4	PLT (RBC)	2
Alanine aminotransferase (ALT)	4	White blood cells (WBC)	2
Aspartate aminotransferase (AST)	3	IL-12 levels of blood	2
Creatinine (CRE)	3	Survival time (days)	2
Urea nitrogen (UBN)	3	Bax (Western blot)	2
PD-1/PD-L1 of tumor	3	Bcl-2 (Western blot)	2

Notably, eight included studies (nine experiments) employed immunodeficient mouse models. Because mechanistic data were limited in these experiments, only tumor-related endpoints were analyzed. Accordingly, all immune-related analyses in this meta-analysis were conducted exclusively in immunocompetent models. APS exhibited tumor-inhibitory effects in both immunocompetent and immunodeficient hosts, suggesting that adaptive immunity may not be the sole contributor to its activity. In nude mouse models, APS increased the Bax/Bcl-2 ratio (Zhou Z. et al., 2017; Qiu et al., 2024), modulated autophagy-related proteins (LC3-II, Beclin-1, p62) (Qiu et al., 2024; Chen et al., 2025), and reduced the expression of drug-resistance transporters (P-gp, MRP, LRP) (Pan, 2020; Zhang Y. et al., 2022), indicating potential involvement of apoptosis induction, autophagy regulation, and enhanced intracellular drug accumulation. Nevertheless, these mechanistic insights should be interpreted cautiously, as they originate from a small number of studies, were synthesized narratively rather than meta-analytically, and often lacked comprehensive pathway-level validation. Therefore, while the dual-pathway model provides a plausible framework, the precise contribution and interplay of immune-dependent and independent mechanisms remain a critical area for future rigorous investigation.

Beyond its potential antitumor mechanisms, APS may also attenuate treatment-related toxicities. In this meta-analysis, APS administration was associated with significantly reduced serum creatinine levels, suggesting possible renal protection. In contrast, hepatic enzymes (AST and ALT) showed no significant alterations, indicating preserved hepatic function. Limited qualitative evidence suggested possible mitigation of chemotherapy-induced myelosuppression and potential hematopoietic support (Li et al., 2018; Pan, 2020). However, because only eight included studies reported safety-related outcomes, the current evidence remains insufficient to draw definitive conclusions.

Overall, APS may exert complementary effects through immune modulation, cytoprotection, and support of host physiological resilience. These observations conceptually parallel the TCM principle of *Fuzheng* (扶正), which emphasizes strengthening host defenses in conjunction with *Quxie* (祛邪) therapies—such as chemotherapy or radiotherapy—to preserve treatment tolerance and improve therapeutic responsiveness.

### 4.4 Limitations and robustness of evidence

Although APS co-administration showed statistically favorable trends across multiple outcomes, the overall robustness of these conclusions is fundamentally constrained by the low quality of the primary studies. All major endpoints were rated as “low” or “very low” certainty by GRADE, reflecting pervasive methodological weaknesses—including inadequate reporting of randomization, allocation concealment, and blinding—that increase the likelihood of biased effect estimates. The substantial and largely unexplained heterogeneity further reduces confidence in pooled results, as effect size variability could stem from uncontrolled differences in APS preparation, dosing, model systems, and experimental procedures. Moreover, publication bias may have inflated positive findings, and data extraction from figures may introduce measurement error. These limitations collectively indicate that the apparent benefits of APS should be interpreted as preliminary rather than definitive. Nonetheless, the generally consistent direction of effects across diverse models provides a tentative signal warranting further investigation. Strengthening the evidence base will require rigorously designed animal studies that adhere to SYRCLE and ARRIVE guidelines.

### 4.5 Implications for clinical translation and future research

To bridge the gap between the preclinical promise of APS and its clinical application, several critical hurdles must be addressed. First, standardized APS preparations need to be developed. This requires both defining biologically relevant chemical attributes (e.g., molecular weight distribution, glycosidic linkage patterns, and monosaccharide composition) and implementing robust quality-control frameworks to ensure batch-to-batch consistency across sourcing, cultivation, extraction, and purification. Second, dosing strategies require systematic optimization. While existing clinical trials have utilized only injectable APS, preclinical data suggest potentially superior efficacy with oral administration. Therefore, developing and rigorously evaluating oral formulations represents a promising future direction—though this necessitates establishing new quality standards and providing mechanistic justification. Third, human safety profiles require careful evaluation. Despite preliminary evidence indicating favorable tolerability and toxicity mitigation in animal models, the evidence remains limited. Future clinical trials should incorporate systematic monitoring for immune-related adverse events (irAEs) and potential drug interactions. In summary, the successful clinical translation of APS in cancer therapy will depend on coordinated advances in standardization, dosing optimization, and comprehensive safety evaluation.

## 5 Conclusion

This meta-analysis provides preclinical evidence that APS may serve as an adjunctive agent to enhance the efficacy of conventional cancer therapies. However, given the low certainty of current evidence, further mechanistic studies and well-designed clinical trials are urgently warranted to establish its efficacy and therapeutic role in oncology.

## Data Availability

The original contributions presented in the study are included in the article/Supplementary Material, further inquiries can be directed to the corresponding authors.
