# Supplementary material for: Synergistic antitumor effects of astragalus polysaccharide: a preclinical systematic review and meta-analysis
Source: Front Pharmacol. 2025 Dec 18;16:1672450. doi: 10.3389/fphar.2025.1672450 (PMC12756362; doi:10.3389/fphar.2025.1672450)
Supplement: Supplementary file 1 [file Table1.docx]

**Supplementary Table 1** Risk of bias and quality assessment of included studies.

| **Study** | **a** | **b** | **c** | **d** | **e** | **f** | **j** | **h** | **i** | **j** | **score** |
| --- | --- | --- | --- | --- | --- | --- | --- | --- | --- | --- | --- |
| Huang et al. 2002 | L | L | U | L | U | U | U | L | L | L | 6 |
| Huo 2016 | L | L | U | L | U | U | U | L | L | L | 6 |
| Zhang et al. 2024 | L | L | U | L | U | U | U | L | L | L | 6 |
| Sha et al. 2022 | L | U | U | U | U | U | U | L | L | L | 4 |
| Wang 2024 | L | L | U | L | U | U | U | L | L | L | 6 |
| Lin et al. 2022 | U | L | U | L | U | U | U | L | L | L | 5 |
| Liu et al. 2024 | L | L | U | L | U | U | U | L | L | L | 6 |
| Lin et al. 2024 | L | L | U | L | U | U | U | L | L | L | 6 |
| Li et al. 2024 | L | L | U | L | U | U | U | L | L | L | 6 |
| Cao et al. 2024 | U | U | U | U | U | U | U | L | L | L | 3 |
| Lv et al. 2017 | L | L | U | L | U | L | U | L | L | L | 7 |
| Zhang 2019 | L | L | U | L | U | U | U | L | L | L | 6 |
| Mu et al. 2019 | L | L | U | L | U | L | U | L | L | L | 7 |
| Song et al. 2022 | L | L | U | L | U | U | U | L | L | L | 6 |
| Huang et al. 2008 | L | L | U | U | U | U | U | L | L | L | 5 |
| Tian et al. 2012 | L | L | U | L | U | U | U | L | L | L | 6 |
| Sun et al. 2013 | L | L | U | U | U | U | U | L | L | L | 5 |
| Ming et al. 2014 | L | L | U | U | U | U | U | L | L | L | 5 |
| Zhuang et al. 2017 | L | L | U | U | U | U | U | L | L | L | 5 |
| Wang et al. 2017 | L | L | U | L | U | U | U | L | L | L | 6 |
| Wang et al. 2017 | L | L | U | U | U | U | U | L | L | L | 5 |
| Zhu et al. 2017 | L | L | U | U | U | U | U | L | L | L | 5 |
| Zhou et al. 2017 | L | L | U | U | U | U | U | L | L | L | 5 |
| Li et al. 2018 | L | L | U | L | U | U | U | L | L | L | 6 |
| Phacharapiyangkul et al. 2019 | U | L | U | L | U | U | U | L | L | L | 5 |
| Bamodu et al. 2019 | L | L | U | L | U | U | U | L | L | L | 6 |
| Pan 2020 | U | L | U | L | U | U | U | L | L | L | 5 |
| Pang 2020 | L | L | U | U | U | U | U | L | L | L | 5 |
| Li et al. 2019 | L | L | U | L | U | U | U | L | L | L | 6 |
| Wang 2021 | U | L | U | L | U | U | U | L | L | L | 5 |
| Zhang et al. 2022 | L | L | U | U | U | U | U | L | L | L | 5 |
| Qu 2022 | L | L | U | U | U | U | U | L | L | L | 5 |
| Gong et al. 2022 | U | L | U | L | U | U | U | L | L | L | 5 |
| Wang 2023 | L | L | U | L | U | U | U | L | L | L | 6 |
| Li et al. 2023 | L | L | U | U | U | U | U | L | L | L | 5 |
| Chen 2024 | L | L | U | U | U | U | U | L | L | L | 5 |
| Qiu et al. 2024 | U | L | U | L | U | U | U | L | L | L | 5 |
| Ma 2024 | L | L | U | L | U | U | U | L | L | L | 6 |
| Sun et al. 2025 | L | L | U | L | U | U | U | L | L | L | 6 |
| Chen et al. 2025 | L | L | U | U | U | U | U | L | L | L | 5 |
| Shi et al. 2025 | L | L | U | U | U | U | U | L | L | L | 5 |

Annotation: H, high risk; L, low risk; U, unclear risk; a, Randomization of sequence generatione (Selectin bias) ; b, Baseline characteristics (Selectin bias); c, allocation concealment (Selectin bias) ; d, random breeding (Performance bias) ; e, Blinding investigators (Performance bias); f, Randomised outcome assessment (Detection bias); g, Blinding of outcome assessors (Detection bias) ; h, Incomplete data reporting (Attrition bias) ; i, Selective results reporting (Reporting bias) ; j, Other sources of bias.

**Supplementary Table 2** Egger's test and trim-and-fill analysis

| **Outcome measures** | **studies** | **Egger's test** | **​Pooled Est (95% CI)​** | | **Trimmed Studies** |
| --- | --- | --- | --- | --- | --- |
|  |  |  | **Original Random** | **Trim and Fill Adjusted** |  |
| Tumor weight | 32 | *t* =-12.89, *P*=0.000 | -0.479 (-0.603, -0.355) | -0.516 (-0.641, -0.399) | 2 |
| Tumor volume | 27 | t =-11.33, *P*=0.000 | -3.004 (-3.713, -2.295) | -3.004 (-3.713, -2.295) | 0 |
| CD8^+^ T cells | 14 | *t* =3.43, *P*=0.005 | 2.143 (1.020, 3.267) | 2.143 (1.020, 3.267) | 0 |
| CD4^+^ T cells | 10 | *t* =2.22, *P*=0.057 | - | - |  |
| spleen index | 10 | *t* =1.31, *P*=0.227 | - | - |  |

**Supplementary Table 3** Meta-regression analysis

| **Parameter** | **Variable** | **Coefficient** | ***t*** | ***P* -value** | **95% CI** |
| --- | --- | --- | --- | --- | --- |
| **Tumor weight** | Species | -0.326 | -0.52 | 0.605 | -1.609, 0.958 |
|  | Model type | -1.271 | -1.19 | 0.243 | -3.453, 0.908 |
|  | Tumor type | -0.143 | -0.46 | 0.651 | -0.788, 0.501 |
|  | Combined therapy | -0.104 | -0.23 | 0.817 | -1.026, 0.817 |
|  | APS Source | -0.324 | -0.33 | 0.742 | -2.333, 1.685 |
|  | Administration route | -0.970 | -1.27 | 0.217 | -2.550, 0.609 |
|  | Treatment initiation | -0.573 | -0.76 | 0.454 | -2.128, 0.981 |
|  | Treatment duration | -0.262 | -0.44 | 0.663 | -1.491, 0.966 |
| **Tumor volume** | Species | 0.995 | 1.44 | 0.167 | -0.455, 2.446 |
|  | Model type | -0.186 | -0.15 | 0.883 | -2.780, 2.406 |
|  | Tumor type | 0.093 | 0.24 | 0.816 | -0.740, 0.928 |
|  | Combined therapy | -0.450 | -0.79 | 0.438 | -1.644, 0.742 |
|  | APS Source | 1.202 | 1.04 | 0.313 | -1.230, 3.635 |
|  | Administration route | -0.803 | -1.03 | 0.315 | -2.438, 0.831 |
|  | Treatment initiation | -0.546 | -0.82 | 0.422 | -1.942, 0.849 |
|  | Treatment duration | -0.432 | -0.65 | 0.523 | -1.825, 0.961 |

**Supplementary Table 4** Quality of evidence based on GRADE’s tool

| **Outcome measures** | **Studies** | **Certainty assessment** | | | | | | **Participants** | | **Effect** | **Quality of the evidence (GRADE)** |
| --- | --- | --- | --- | --- | --- | --- | --- | --- | --- | --- | --- |
|  |  | **Study design** | **Risk of bias** | **Inconsistency** | **Indirectness** | **Imprecision** | **Others** | **Com** | **Mon** | **Absolute**  **(95% CI)** |  |
| Tumor weight | 32 | Randomised trials | serious^a^ | not serious | serious^c^ | not serious | publication bias strongly suspected^e^ | 221 | 220 | SMD = -2.38 [-2.95, -1.82] | ⨁◯◯◯ Very low |
| Tumor volume | 27 | Randomised trials | serious^a^ | not serious | serious^c^ | not serious | publication bias strongly suspected^e^ | 197 | 196 | SMD = -2.70 [-3.36, -2.04] | ⨁◯◯◯ Very low |
| Spleen index | 10 | Randomised trials | serious^a^ | serious^b^ | serious^c^ | not serious | none | 88 | 87 | SMD = 1.70 [0.79, 2.61] | ⨁◯◯◯ Very low |
| Thymus index | 8 | Randomised trials | serious^a^ | not serious | serious^c^ | not serious | none | 63 | 62 | SMD = 1.82 [0.76, 2.88] | ⨁⨁◯◯ Low |
| Lung metastatic nodules | 4 | Randomised trials | serious^a^ | not serious | serious^c^ | not serious | none | 20 | 20 | SMD = -3.67 [-5.74, -1.60] | ⨁⨁◯◯ Low |
| Survival time (days) | 2 | Randomised trials | serious^a^ | not serious | serious^c^ | not serious | none | 12 | 12 | MD = 10.31 [6.11, 14.51] | ⨁⨁◯◯ Low |
| CD8+ T cells | 14 | Randomised trials | serious^a^ | not serious | serious^c^ | not serious | publication bias strongly suspected^e^ | 82 | 82 | SMD = 1.87 [0.82, 2.93] | ⨁◯◯◯ Very low |
| CD4+ T cells | 10 | Randomised trials | serious^a^ | serious^b^ | serious^c^ | not serious | none | 63 | 63 | SMD = 1.57 [0.41, 2.72] | ⨁◯◯◯ Very low |
| TNF-α levels | 7 | Randomised trials | serious^a^ | not serious | serious^c^ | not serious | none | 63 | 62 | SMD =8.96 [4.96, 12.95] | ⨁⨁◯◯ Low |
| IL-2 levels | 7 | Randomised trials | serious^a^ | serious^b^ | serious^c^ | not serious | none | 67 | 66 | SMD = 4.54 [1.57, 7.51] | ⨁◯◯◯ Very low |
| IFN-γ levels | 6 | Randomised trials | serious^a^ | not serious | serious^c^ | not serious | none | 48 | 47 | SMD = 4.29 [1.80, 6.78] | ⨁⨁◯◯ Low |
| IL-10 levels | 4 | Randomised trials | serious^a^ | serious^b^ | serious^c^ | not serious | none | 41 | 41 | SMD = -8.47 [-16.71, -0.23] | ⨁◯◯◯ Very low |
| IL-1β levels | 4 | Randomised trials | serious^a^ | serious^b^ | serious^c^ | not serious | none | 37 | 37 | SMD = 5.22 [1.89, 8.55] | ⨁◯◯◯ Very low |
| IL-6 levels | 4 | Randomised trials | serious^a^ | serious^b^ | serious^c^ | not serious | none | 41 | 41 | SMD = 11.07 [1.44, 20.70] | ⨁◯◯◯ Very low |
| TGF-β levels | 4 | Randomised trials | serious^a^ | serious^b^ | serious^c^ | serious^d^ | none | 37 | 37 | SMD = -1.96 [-5.16, 1.24] | ⨁◯◯◯ Very low |
| IL-12 levels | 3 | Randomised trials | serious^a^ | not serious | serious^c^ | not serious | none | 21 | 21 | SMD = 3.66 [2.53, 4.80] | ⨁⨁◯◯ Low |

**Supplementary Table 4** (*continued*) Quality of evidence based on GRADE’s tool

| **Outcome measures** | **Studies** | **Certainty assessment** | | | | | | **Participants** | | **Effect** | **Quality of the evidence (GRADE)** |
| --- | --- | --- | --- | --- | --- | --- | --- | --- | --- | --- | --- |
|  |  | **Study design** | **Risk of bias** | **Inconsistency** | **Indirectness** | **Imprecision** | **Others** | **Com** | **Mon** | **Absolute(95% CI)** |  |
| ALT | 4 | Randomised trials | serious^a^ | not serious | serious^c^ | serious^d^ | none | 28 | 28 | SMD = -1.65 [-3.36, 0.06] | ⨁◯◯◯ Very low |
| AST | 3 | Randomised trials | serious^a^ | serious^b^ | serious^c^ | serious^d^ | none | 22 | 22 | SMD = -0.70 [-2.32, 0.93] | ⨁◯◯◯ Very low |
| CRE | 3 | Randomised trials | serious^a^ | not serious | serious^c^ | not serious | none | 22 | 22 | SMD=-1.15 [-1.82, -0.48] | ⨁◯◯◯ Very low |
| UBN | 3 | Randomised trials | serious^a^ | not serious | serious^c^ | serious^d^ | none | 22 | 22 | SMD = -0.90 [-2.01, 0.21] | ⨁◯◯◯ Very low |
| PD-1/PD-L1 | 3 | Randomised trials | serious^a^ | serious^b^ | serious^c^ | not serious | none | 35 | 35 | SMD= -3.66[-6.11, -1.20] | ⨁◯◯◯ Very low |

CI: confidence interval; MD: mean difference; SMD: standardised mean difference; a: Most studies did not report transparently the generation of random sequences and the implementation of blinding; b: Conflicting effect estimates across included studies or results not robust in sensitivity analysis; c: Studies using lower species animal models may not fully recapitulate human pathophysiology; d: The 95% confidence interval crosses the line of no effect; e: Egger's test *P* < 0.05

**Supplementary Table 5** Inclusion and exclusion criteria

| **Category** | **Inclusion Criteria** | **Exclusion Criteria** |
| --- | --- | --- |
| **Population** | Animal cancer models | Clinical, in vitro, or non-cancer disease models |
| **Intervention** | APS combined with conventional therapy | APS monotherapy; crude or multi-herbal extracts |
| **Comparison** | Conventional therapy alone | No-treatment or non-standard therapy controls |
| **Outcomes** | Quantifiable antitumor efficacy or biomarker data | Qualitative or non-quantifiable outcomes |
| **Study Design** | Randomized controlled animal trials | Non-original research; lack of independent control group |
| **Other** | English or Chinese publications with extractable data | Other languages publications without accessible translations; duplicate publications; incomplete data |
